# Occurrence of *Macrophomina phaseolina* on Chickpea in Italy: Pathogen Identification and Characterization

**DOI:** 10.3390/pathogens11080842

**Published:** 2022-07-27

**Authors:** Eliana Dell’Olmo, Pasquale Tripodi, Massimo Zaccardelli, Loredana Sigillo

**Affiliations:** Council for Agricultural Research and Economics, Research Centre for Vegetable and Ornamental Crops, via Cavalleggeri 25, 84098 Pontecagnano, Italy; eliana.dellolmo@crea.gov.it (E.D.); pasquale.tripodi@crea.gov.it (P.T.); massimo.zaccardelli@crea.gov.it (M.Z.)

**Keywords:** *Macrophomina phaseolina*, chickpeas, pathogenicity, phylogenesis

## Abstract

Climate change has led to the spread of plant pathogens in novel environments, causing dramatic crop losses and economic damage. *Botryosphaeriaceae* represents a massive fungal family, containing a huge number of plant pathogens, which are able to infect several hosts. Among them, *Macrophomina phaseolina* is a necrotrophic fungus, responsible for several plant diseases, including the soft stem rot of common bean, crown rot on strawberry and charcoal rot of several legumes. Here, *Macrophomina,* causing crown charcoal rot in chickpeas, was isolated from symptomatic plants in Cicerale (SA), Campania, South Italy. Morphological and molecular characterization was carried out and pathogenicity tests were performed. Phylogenetic analyses were performed comparing *Macrophomina* strains coming from different geographic areas and hosts. The experiments confirmed the pathogenicity of the isolate CREA OF 189.2 on chickpea, while host range highlighted the polyphagous nature of this strain; thus, symptoms were reported on lentils, common bean and cantaloupe. The multidisciplinary approach allows us to increase the knowledge about this emerging pathogen. To the best of our knowledge, this is the first report on *Macrophomina phaseolina* from chickpeas in Italy.

## 1. Introduction

One of the most observed phenomena linked with climate change is the spread of plant pathogens, originally confined to specific ecological niches [1]. The introduction of novel pathogens in agriculture systems, in which they are not present so far, opens the way to undiscovered problems, beyond the loss of yields and related to the ineffectiveness of the normal control system employed to fight the infections [2]. In this scenario, fungi belonging to *Botryosphaeriaceae* are worthy of mentioning since they represent a huge family, including necrotrophic fungi [3], which have been found in different areas of tropical and temperate regions. The *Macrophomina* genus is included in the *Botryosphaeriaceae* family and represents one of the most spread pathogens all over the globe; thus, it is able to infect more than 700 plant species, including economically relevant crops [4,5]. As the other components of *Botryosphaeriales*, *Macrophomina* is a necrotrophic fungal pathogen considered manly soilborne [6], due to its ability to survive for several years as microsclerotia, but evidence suggests that it is also able to be preserved in the seeds acting as a seedborne pathogen [4]. Moreover, the ability of *Macrophomina* to infect the hosts in each stage of growth has been reported [7] and it has been highlighted that the fungus attacks different part of the plants, generating different symptoms, such as dry root rot, charcoal rot and soft stem rot [8,9]. The devastating effects of *Macrophomina* on hosts, derived from its ability to interfere with the transport of nutrients and water from the roots to the upper part of the plants, induces the early seedlings’ death [4]. Efforts have been made to better understand *Macrophomina* behavior; phylogenetic studies aiming to distinguish genetic clade [10] based on host or geographical location were unsuccessful [11]. Additionally, due to the polyphagous nature of *Macrophomina*, it has been isolated from a vast range of plants, including food crops, such as maize and sorghum, fiber crops, such as jute and cotton [12], but also from oil crops (sunflower and soybean) [13], strawberry [14], watermelon [15], pepper [16] and legumes, such as common bean (*Phaseolus vulgaris*), soybean (*Glycine max* L.), mungbean (*Vigna radiata* (L.) R. Wilczek) and chickpea (*Cicer arietinum* L.) [17], causing dramatic economic losses. The preference of *Macrophomina* for warm climate and low water stress, along with the wide host range, shed a light on the crucial role of this pathogen. The increasing production of legumes for human consumption in Europe, particularly in Italy, with the average cultivated area of 5265 ha for beans and 17,617 ha for chickpeas, has made urgent the observation of *Macrophomina* spread on this territory [18]. In this work, the causal agent of chickpea crown and root charcoal rot was isolated. Morphological and molecular characterization was performed, and the pathogenicity assessed on different plant hosts.

## 2. Results

### 2.1. Isolation and Morphological Identification

Plant samples showed serious damage due to the presence of yellowed, dried and dead plants, with a total incidence of 80%.

**Figure 1:**
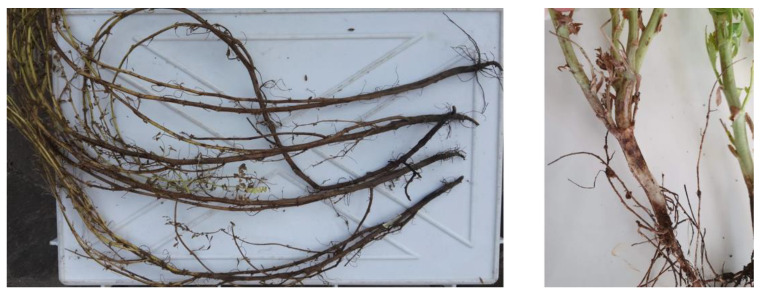
Chickpea plants showing crown charcoal dry rot and root rot.

In a more careful visual analysis, severe desiccation of the aerial part was recorded, while crown and roots appeared dry and black and secondary roots were almost absent (Figure 1). 

After 7 days, *M. phaseolina* was uniformly isolated from all the symptomatic samples. In order to identify the isolates, monohyphal colonies were grown on fresh PDA for 10 days. In these conditions, *M. phaseolina* isolates from chickpea show dark gray and flat colonies in the early stage of growth, which became darker with aging. The colonies were black on the reverse side of the plate and the morphology on PDA is comparable with the reference *M. phaseolina* CREA OF 373.2 (Supplementary Figure S1a,d). Further observations were carried out by using optical microscopy, which revealed the presence of septate dark hyphae with branches, placed at 90° over the leading hypha (Figure S1b,e). Moreover, the connections between the main hyphae and the branches were characterized by a constriction and each was followed by a septum, which is a hallmark for this class of fungi. The analyses carried out on microsclerotia highlighted that the CREA OF 189.2 isolate has circular microsclerotia, with an average width of 79.3 µm (ranging from 72.4 to 85.0 µm), while the reference strain CREA OF 373.2 produces ovoid microsclerotia, with an average width of 97 µm (ranging from 93.0 to 99.2 µm) (Figure S1c,f). Finally, differences between the isolates were also observed in the number of microsclerotia, with CREA OF 189.2 having 50% less microsclerotia than the reference strain. These findings perfectly agree with the evidence collected until now about the high variability among *Macrophomina* strains. One isolate, coded as CREA OF 189.2, was used for further characterizations.

### 2.2. Molecular Characterization of Macrophomina phaseolina CREA OF 189.2

The molecular analyses of *Macrophomina* isolates were carried out by using the species-specific primers employed by Santos et al. 2020, designed on the calmodulin (*CAL*) and translation elongation factor (*Tef1-α*) genes. Besides, to be highly specific for the *Macrophomina* genus, this set of primers allows one to discriminate between three different species of the pathogen: *M. phaseolina* (Mp), the most common species, from *M. pseudophaseolina* (Ms) and *M. euphorbiicola* (Me), which is the least spread with respect to others. Accordingly, with the previous findings, CREA OF 189.2 was found to be positive at the amplification with CAL primers for Mp and Me, confirming the conservative nature of the region, which make the discrimination between the species incomplete. By contrast, the amplification performed with primers designed on the *Tef 1-α* gene showed better ability to discriminate between the species, giving a positive result only when the isolates from chickpeas were amplified with MpTef primer pairs, confirming the identity of the strain as *M. phaseolina* (Figure S2). In this analysis, the strain *M. phaseolina* CREA OF 373.2 was used as reference. The ITS and *Tef 1-α* regions were sequenced, using ITS1/4 and Tef_728-986_ primers, and the sequences were deposited in GenBank under the accession numbers: ON063435 and ON181257. According to the BLASTn analysis, CREA OF 189.2 showed 100% identity (coverage 99–100%) with several annotated *Macrophomina phaseolina* ITS and *Tef-1α* sequences. 

### 2.3. Pathogenicity of M. phaseolina CREA OF 189.2 Isolate from Chickpea

**Figure 2:**
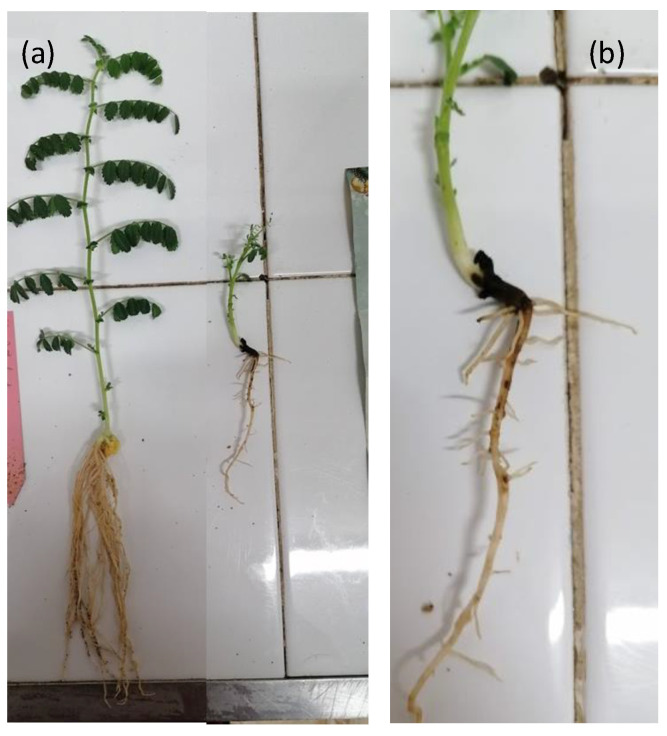
Symptoms caused in plants by the artificial inoculation of *M. phaseolina* CREA OF 189.2 of chickpea seeds. (**a**) Control (right side) and inoculated (left side) chickpea plants; (**b**) detail of charcoal crown rot in infected chickpea plants.

The pathogenicity of CREA OF 189.2 was tested on chickpea seedlings through inoculation of the seeds. Briefly, they were put in contact with one-week-old fungus for 24 h and then, sowed into sterile sand. The first symptoms of chickpea strain infection were observed a few days after sowing. Moreover, a 90% reduction in seed germination was reported compared to the non-inoculated control (Figure 2a). Ten days after inoculation, the seedlings that survived showed the initial symptoms of necrosis at the crown and the discoloration of the developed roots. At 15 days, typical lesions of the charcoal crown rot were clearly visible and became most severe during the days, the root rot developed, and all the affected plants showed reduced dimensions with respect to the non-inoculated control (Figure 2b). In order to fulfill Koch’s postulates, the isolation of the pathogen was performed from inoculated and symptomatic plants, which confirmed the presence of *Macrophomina* sp. in all the affected tissues. Conversely, the pathogen was not isolated from the non-inoculated control plants. As a reference for typical charcoal rot symptoms, tests were conducted on common bean (*Phaseolus vulgaris*) by using the isolate CREA OF 373.2 (Figure S3). This strain showed the damping rot at the stem level, which spread up throughout the plant, causing the development of black spot with sharp margins. The progression of the infection leads to wilting and, ultimately, the death of the infected common bean plants. The results highlighted differences in symptoms, which may vary depending on the host plant and fungal strain. 

### 2.4. Host Range of M. phaseolina CREA OF 189.2

**Figure 3:**
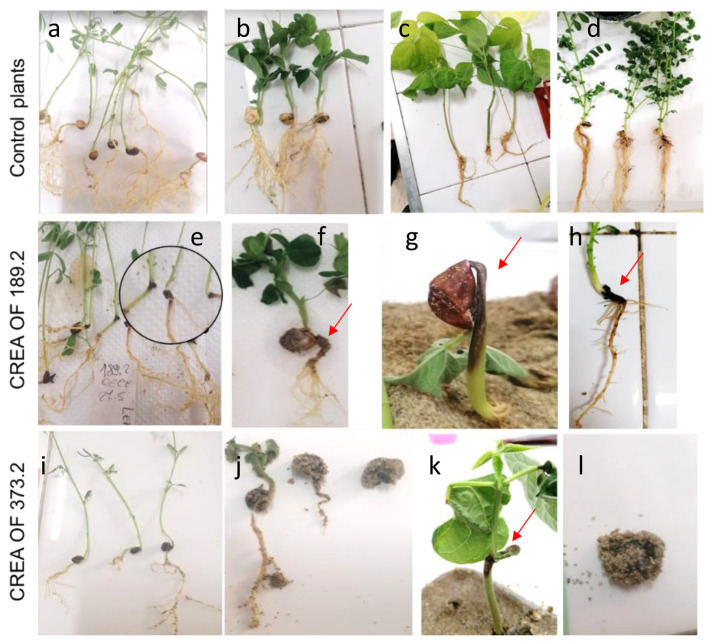
Disease symptoms caused by *M. phaseolina* CREA OF 189.2 and CREA OF_373.2 isolates on different legume species. First row: non-inoculated plants (**a**–**d**); second row: lentils, pea, bean, and chickpea plants inoculated with CREA OF 189.2 isolate (**e**–**h**); third row: lentils, pea, bean and chickpea inoculated with the CREA OF 373.2 isolate (**i**–**l**). The circle in the “e” box highlights details of symptoms on lentils.

One of the main concerns about *Macrophomina* species regards its polyphagous nature and the ability to inhabit several ecological niches, which make the pathogen even more dangerous for crops. Therefore, different species of common crops were employed in a wide host range experiment, including legumes, such as common bean, peas and lentils, solanaceous species, such as tomato and pepper, and cucurbitaceous species, such as melon. Legume plants inoculated with CREA OF 189.2 showed different types of symptoms in several stages of plant development, with respect to the control plants that showed no symptoms (Figure 3a–d). 

Lentils were not affected at the seed level but, upon 7 days from the sowing, crown dry rot symptoms were reported as linear necrotic lesions with consequent yellowing of the areal part of the plants (Figure 3e). Additionally, during the incubation time, the crown rot symptoms progressed along with foliage damage (DI = 51.6%). A different scenario was reported on inoculated peas (Figure 3f), which showed an average germination rate of 20% with respect to those of the control plants (85%) (Figure 3b) and, after 18 d, surviving pea plants displayed symptoms of root rot and dwarfing. On common bean, which was the main host for *Macrophomina* spp., a low germination rate (average 3.3%) or typical symptoms of stem dry rot were observed (Figure 3g,k). Similar symptoms were observed for the reference strain CREA OF 373.2 (Figure 3i–l), although symptoms on lentil were less severe with respect to those caused by the CREA OF 189.2 isolate.

**Figure 4:**
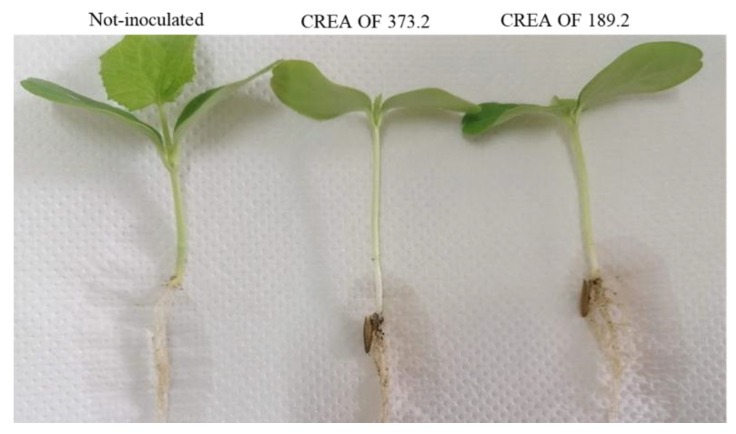
Symptoms of CREA OF 189.2 and CREA OF 373.2 isolates compared with the non-inoculated control plants.

On the other hand, 18-days post-inoculation, melon plants showed symptoms of a slight crown rot and plant growing reduction (Figure 4). Differently, when CREA OF 189.2 was tested on pepper species, a dramatic effect on seeds was reported, with the complete inhibition of germination, in these experimental conditions (data not shown). Finally, CREA OF 189.2 was tested in tomato plants with no effects, at least on this variety and in the conditions used for the in vivo assay. In order to compare the results obtained for the CREA OF 189.2 isolate, assays were also performed with the collection strain CREA OF 373.2. Interestingly, the latter cause germination inhibition in chickpea, pea (Figure 3j,l), pepper (average percentage of germination: 3.3, 8 and 0, respectively) and cantaloupe, where crown rot was also caused on the seedling (Figure 4). Instead, a different trend was reported for lentils, which showed no symptoms of infection when inoculated with CREA OF 373.2 (Figure 3i), suggesting differences between the strains. The obtained results confirm the polyphagous behavior of the *Macrophomina* pathogen, with a severe impact on a massive range of economically important crops. 

### 2.5. Phylogenetic Analysis of M. phaseolina CREA OF 189.2 Isolate

**Figure 5:**
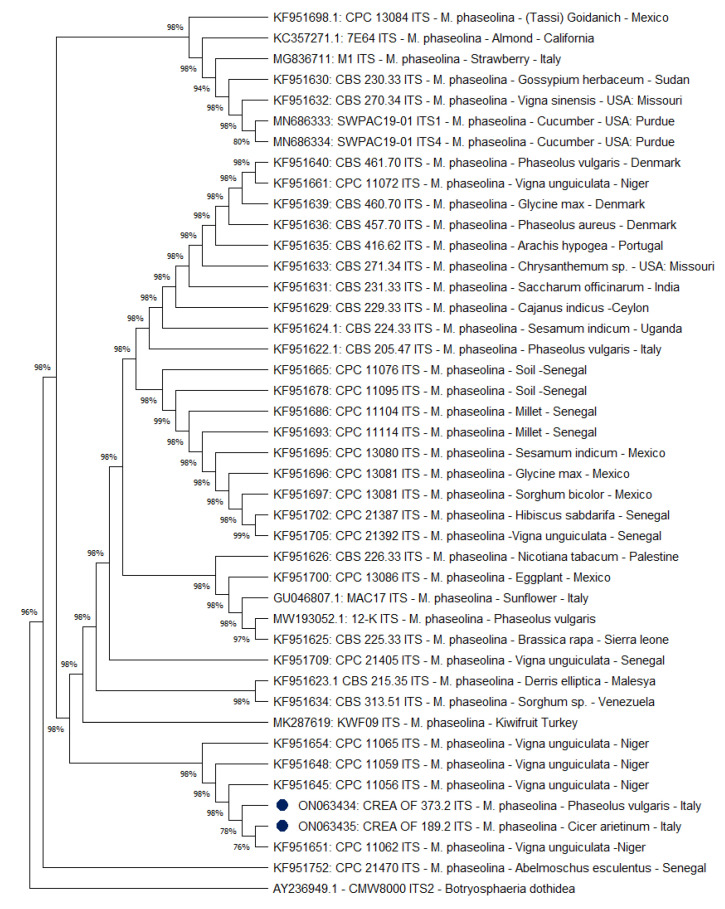
Phylogenetic analysis based on ITS sequences of 42 *Macrophomina phaseolina* isolates. ITS2 sequence of *Botryosphaeria dothidea* CMW8000 was used as the outgroup. The bootstrap tree inferred from 1000 replicates is shown. The phylogenetic tree was inferred by using the maximum likelihood method and Tamura–Nei model. The percentage of replicate trees in which the associated taxa clustered together in the bootstrap test is shown next to the branches. The tree is drawn to scale, with branch lengths measured in the number of substitutions per site (next to the branches). Blue dots indicate the *Macrophomina* strains isolated in Italy. Phylogenetic analyses were conducted in MEGA X.

To further characterize the CREA OF 189.2 strain, phylogenetic analyses were carried out to verify a correlation between the host species, the geographic area, and the pathogen. In this context, a selection of 42 GenBank accessions of *M. phaseolina* ITS sequences, derived from all five continents and isolated from different plant hosts, was used to build a phylogenetic tree by using the maximum likelihood method. The ITS analysis revealed that the isolates CREA OF 189.2 and CREA OF 373.2 were grouped closely and in a cluster, including *Vigna unguiculata* from Niger as a host. (Figure 5). Few differences were observed among the *M. phaseolina* isolates that constitute a unique group (96%), with respect to the outgroup species, *B. dothidea* CMW8000.

**Figure 6:**
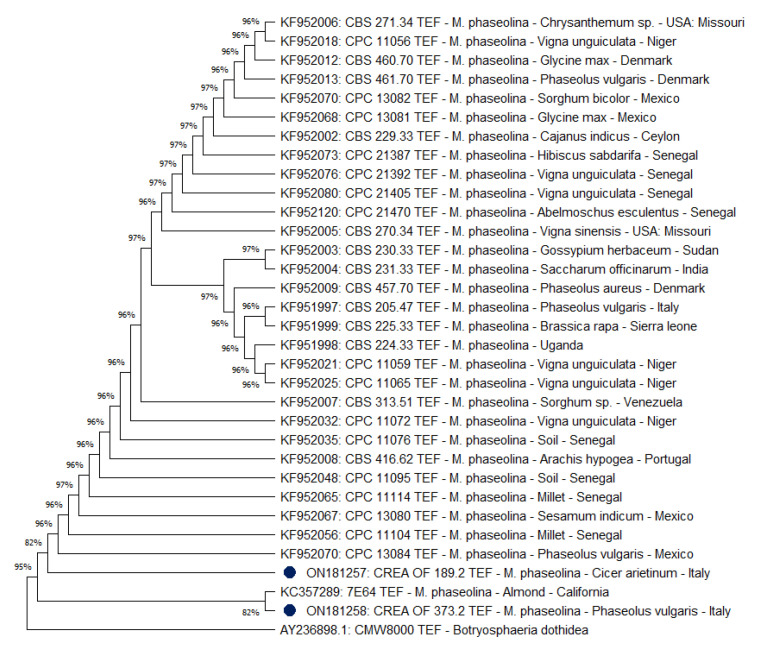
Phylogenetic analysis obtained from the *Tef* 1-α sequences. The phylogenetic tree was inferred by using the maximum likelihood method and Tamura–Nei model. The bootstrap tree inferred from 1000 replicates is shown. The percentage of replicate trees, in which the associated taxa clustered together in the bootstrap test, is shown next to the branches. The tree is drawn to scale, with branch lengths measured in the number of substitutions per site (next to the branches). Blue dots indicate the *Macrophomina* isolates from Italy. This analysis involved 32 *Macrophomina phaseolina Tef* 1-α sequences. TEF sequence of *Botryosphaeria dothidea* CMW8000 was used as the outgroup. Phylogenetic analyses were conducted in MEGA X.

Subsequently, thirty *Tef 1-α* sequences, selected with the same criteria used before, were included in a phylogenetic analysis. The results (Figure 6) confirmed that isolates CREA OF 189.2 and CREA OF 373.2 were clustered closely, although a greater degree of separation was found within this region. As for ITS, few differences were observed among the *M. phaseolina* isolates, showing a unique group at 95% of similarity with respect to the outgroup *B. dothidea* CMW8000. In both instances, the close relationship with strains isolated from other species with different origins suggests a possible taxonomic-based host affinity rather than an association between genotype and geographic location of the isolates.

**Figure 7:**
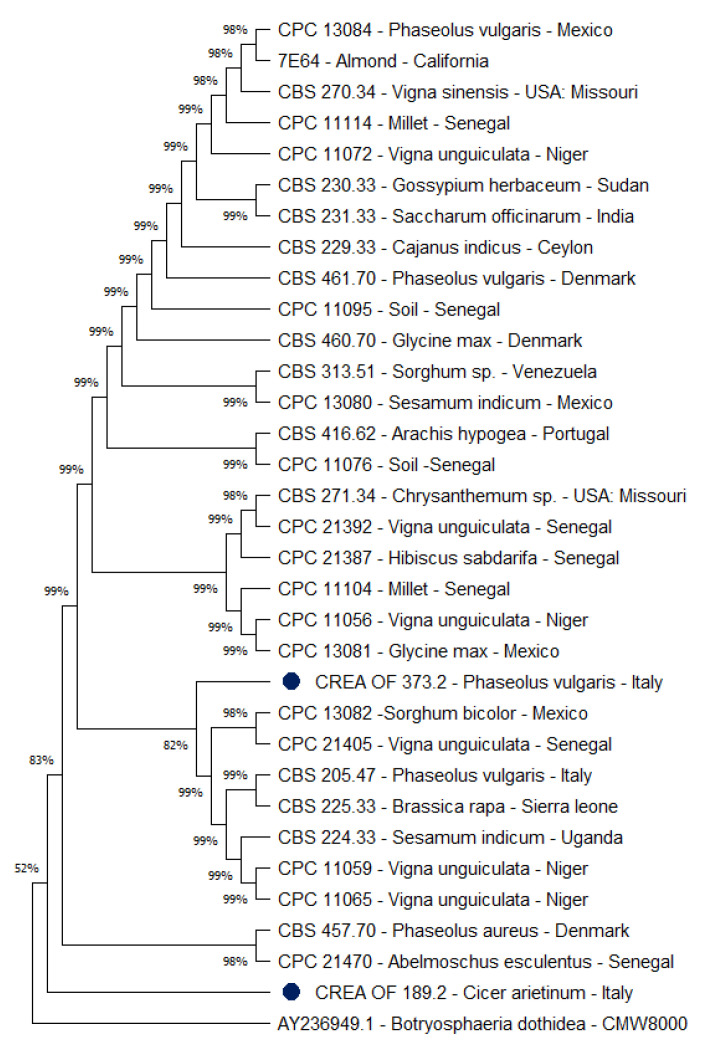
Combined tree of ITS and *Tef 1-α* region. The phylogenetic tree was inferred by using the maximum likelihood method and Tamura–Nei model. The bootstrap tree inferred from 1000 replicates is shown. The percentage of replicate trees, in which the associated taxa clustered together in the bootstrap test, is shown next to the branches. The tree is drawn to scale, with branch lengths measured in the number of substitutions per site (next to the branches). Blue dots indicate the *Macrophomina* isolates from Italy. *Botryosphaeria dothidea* CMW8000 was used as the outgroup. Phylogenetic analyses were conducted in MEGA X.

For the isolates for which both the ITS and *Tef* 1-α sequences are deposited, a concatenated phylogenetic tree was built. The analysis allowed to determine a slightly higher dissimilarity between CREA OF 189.2 and the other accessions (Figure 7). Except for CREA OF 189.2, all isolates showed a high level of similarity up to 99%.

In addition, all *M. phaseolina* isolates produced a unique group (83% of similarity) with respect to the outgroup species. The differences observed seem to be specific for the isolate, without relation to the host and the geographic location.

## 3. Discussion

*Botryosphaeriaceae* is a fungal family comprising almost 500 species of plant pathogens [19], including *M. phaseolina*, the causal agents of charcoal rot in common bean [20] and other legumes, as well as root rot [8,9]. Nevertheless, this pathogen has been isolated from different plant species, such as: strawberry, peanut and basil [14,21,22]. The wide range of infected hosts and the ubiquitous geographic distribution of *M. phaseolina* make it one of the most studied emerging pathogens all over the globe. In this context, the present work focused on *M. phaseolina* isolates obtained from symptomatic chickpea plants, collected in Cicerale (SA), Campania (Southern Italy). The isolate CREA OF 189.2 was selected as a representative and its morphological features were typical of *Macrophomina* spp., due to a dark and flat colony [23], septate dark mycelia, with a bottle neck between the main hypha and the right angle branches, and microsclerotia are black and ovoid or circular, as previously reported [3]. Species-specific primers, designed on two conserved loci *CAL* and *Tef 1-α*, were used for the identification of isolate and to discriminate between *Macrophomina* species. The amplification patterns agreed with those previously obtained by Santos et. al., 2020 [24], identifying the isolate CREA OF 189.2 as *M. phaseolina*, which showed the same PCR pattern obtained for the reference isolate (CREA OF 373.2). A further molecular analysis was performed by amplifying and sequencing the ITS and the *Tef 1-α* regions, followed by BLASTn. The multiple alignment in the GenBank database revealed the correspondence of the CREA OF 189.2 isolate with several *M. phaseolina* sequences, confirming the identification obtained with the species-specific primers. When the CREA OF 189.2 isolate was tested on chickpeas, it confirmed its ability to inhibit germination. Moreover, the isolate causes charcoal dry rot of crown and root rot in surviving seedlings, as already observed in cowpeas, peanut and cotton [11,25]. Therefore, in the host range experiments, *M. phaseolina* CREA OF 189.2 confirms its ability to infect other legumes, inducing charcoal crown rot in lentil, seed and root rot, in pea and stem rot in common bean, consistent with several studies indicating different symptomatology, depending on the host [8,17]. On the other hand, variable behavior was reported on solanaceous and cucurbitaceous crops; indeed, symptoms in the seeds were observed in pepper, while slight crown rot was reported in melon. Finally, on tomato, no symptoms were recorded, probably due to the variety used, to the isolate or to the experimental conditions. This evidence confirms the polyphagous nature of *M. phaseolina* CREA OF 189.2, as reported before for the species. Finally, phylogenetic analyses were performed, based on ITS and *Tef* region of different *M. phaseolina* strains isolated in several geographic locations and in a different range of host plant species. 

Based on the resulting ITS/*Tef 1-α*-gene tree (Figure 7), isolates could not be allocated to specific groups according to host or geographic origins, confirming the results of other studies focused on high genetic variability in *M. phaseolina* [11,26,27,28,29].

To our knowledge, this is the first report of *M. phaseolina* isolated from chickpea in Italy, representing a potential threat for chickpea production in the coming years.

## 4. Materials and Methods

### 4.1. Plant Material and Fungal Isolation

Isolates were retrieved in June 2020 obtained from a naturally infected chickpea field in the Cicerale area (Salerno province, Campania region, Italy). Symptomatic plants were collected and transported to the Diagnostic Laboratory of CREA Research Centre for Vegetable and Ornamental Crops, in Pontecagnano (SA), Italy. 

Leaves and stems were cut off and basal part was surface sterilized by soaking in NaClO for 20 min while shaking. Then, samples were washed three times in sterile distilled water and left to dry under sterile hood. Two three-millimeter pieces from dark rot tissues to a healthy white one was placed on Potato dextrose agar (PDA) plates supplemented with 100 µg/mL Chloramphenicol, 50 µg/mL Streptomycin and 50 µg/mL Neomycin and finally incubated at 24 °C for 7 d. The developed isolates CREA OF 189.1, 189.2 and 189.3, were conserved in 30% glycerol at −80 °C and used for further experiments.

### 4.2. Morphological Characterization of M. phaseolina Isolates

Fungal isolates were grown on PDA plates for 7–10 d at 25 °C in the dark. The colonies were described by following the general features of *M. phaseolina*, in terms of colony growth, colony and mycelium morphology [30,31,32,33]. Thus, the isolates, which showed the typical features of *Macrophomina* sp., were further characterized by microscopic analyses. Nikon eclipse 90i microscope was used with 20 or 40× magnification, in transmitted light configuration. Measurements were carried out on 10 hyphae and 10 microsclerotia and the average values were reported. Morphological characteristics were reported such as mycelium color, septa and right-angle hyphae [19]; moreover, the presence of microsclerotia was evaluated along with shape, color, dimensions and abundance [30]. The strain CREA OF 373.2, isolated from common bean in the same year, which showed the typical *M. phaseolina* stem soft rot symptoms and stored in the internal CREA collection, was used as reference strain in all the experiments. The measurements are the mean of three independent replicates.

### 4.3. Molecular Identification of M. phaseolina CREA_OF Isolates

Monohyphal culture of *M. phaseolina* was transferred on PDA and grown for 7 days at 25 °C. Then, agar slant of 5 mm × 5 mm was inoculated in Potato Dextrose Broth (PDB, Difco) and incubated at 25 °C for 72 h, by rotary shaking. The mycelia were recovered by filtration through sterile filter gauze and the biomass was collected into a mortar and ground in liquid nitrogen. The DNA extraction was carried out by using a Genomic DNA isolation kit (Norgen, Biotek Corp., Thorold, ON, Canada), following the manufacturer instructions. The PCR amplification was performed by using the specific primers MpCal/MpTef, MsCal/MsTef and MeCal/MeTef designed by Santos and co-workers [24]. The PCR thermal cycles were set to an initial denaturation at 95 °C for 2 min, followed by 35 cycles at 95 °C for 60 s, 68 °C for 30 s and 72 °C for 60 s; the final extension was at 72 °C for 10 min. To confirm the results obtained with the species-specific primers, the conservative Internal Transcribed Spacer ITS region and the Translation Elongation Factor *Tef 1-α* were amplified by using Phusion™ High-Fidelity DNA Polymerase (2 U/µL) according to manufacturer instructions and sequenced. Amplicons were then purified mixing 5 μL of PCR product with 2 μL of ExoSAP-IT (Thermofisher, Foster City, CA, USA), followed by incubation at 37 °C for 4 min, inactivation at 80 °C for 1 min. Sequencing reaction was prepared with Big Dye terminator v3.1 Cycle sequencing kit (Thermofisher, Foster City, CA, USA) and amplification cycle was set as following: denaturation at 96 °C for 1 min, 25 cycles of 96 °C for 10 s, 50 °C for 5 s and 60 °C for 2 min, holding at 4 °C. Sequencing reactions were then purified using the X-Terminator Purification Kit according to manufacturer’s instruction and analyzed on an automated SeqStudio™ Genetic Analyzer sequencer (Thermofisher, Waltham, MA, USA). Base Calling was performed using SeqScape® v2.0 (Thermofisher, Waltham, MA, USA). Sequences were trimmed and manually edited using Chromas Lite. Sequences were identified using a nucleotide Basic Local Alignment Search Tool (BLAST) and then deposited in GenBank with the following accession numbers: CREA OF 189.2 ITS ON063435, CREA OF 373.2 ITS ON063434, CREA OF 189.2 *Tef 1-α* ON181257 and CREA OF 373.2 *Tef 1-α* ON181258. 

### 4.4. Pathogenicity Test of M. phaseolina CREA OF 189.2 on Chickpea and Host Range

In order to investigate the pathogenicity of CREA OF 189.2, pathogenicity tests were performed. Chickpea seeds were preventively sterilized with sodium hypochlorite (1% *v*/*v*) for 5 min, washed with sterile distilled water and then, dried on sterile filter paper under hood. Seeds (20 for each treatment) were put on the mycelium of CREA OF 189.2 and CREA OF 373.2 10-day-old PDA colonies and incubated at 26 °C for 24 h with 12-h photoperiod [31,32]. The negative control consisted of seeds exposed to sterile PDA under the same conditions; the experiments were carried out in duplicate. Afterwards, infected seeds were transferred in sand, previously sterilized twice at 120 °C for 20 min, placed in plastic boxes (11 cm × 11 cm × 3.5 cm) and incubated in a temperature-controlled room at 26 °C ± 2 °C and with manual irrigation, when necessary. The evaluation of the pathogenicity was based on the number of emerged plants and the number of diseased seedlings observed after 20 d. The rotting of the seeds and blacking of crown and roots were recorded. The germination rate was calculated as the percentage of germinating seeds on the total of sown seeds.

The host range experiments were carried out on common bean (*Phaseolus vulgaris*) cv. Dente di morto retrieved from CREA seed collection, lentil (*Lens culinaris Medk*) local variety Lenticchia di Valle agricola, pea (*Pisum sativum*) cv. Pisello A Grano Rugoso Tondo and tomato (*Solanum lycopersicum*) cv. Gianna obtained from Blumen Group S.p.A., (Milano, Italy). Pepper (*Capsicum annuum*) cv. Giallo d’Asti, obtained from Teraseeds s.r.l. cons (Gambettola (FC), Italy) and cantaloupe (*Cucumis melo* ) cv. Retato Degli Ortolani, from Pagano Costantino and F.lli s.r.l., Scafati (SA), Italy. The same protocol described for the pathogenicity test was used and the symptoms were recorded as presence/absence of damage. In the case of lentils, the following disease scale was used: 0 = no symptoms, 1 = surface necrosis on the crown and early leaf yellowing, 2 = deep necrosis on crown, blacking of roots and foliage wilting, 3 = dead plants. The data were elaborated by calculating the disease index as DI = (0A + 1B + 2C + 3D) * 100/((A + B + C + D) * 3), where:

A, B, C and D were number of plants that showed the symptom level 0, 1, 2 and 3, respectively. 

The experiments were carried out in triplicate.

### 4.5. Phylogenetic Analysis of Macrophomina Phaseolina CREA_OF_189.2

Phylogenetic analyses were conducted on the CREA OF 189.2 isolate, starting by editing the sequences using MEGA X software [33]. After that, forty-two ITS and thirty-two *Tef1-α* sequences derived from GenBank were aligned using the default settings of Muscles and trimmed [34]. The obtained alignment was employed to construct a max likelihood tree using the ITS and TEF sequences separately or merged, to obtain a concatenated tree. Once concatenated, sequences were re-aligned. All obtained alignments were employed to construct the respective phylogenetic trees using max likelihood method and Tamura–Nei model with 1,000 bootstrap replicates using MEGA X (Table 1).

## 5. Conclusions

In conclusion, this work reports, for the first time, *M. phaseolina* on chickpea in Italy. The characterization of the pathogen contributes to knowledge regarding the infection and effective control strategies. Moreover, the evidence about the seed transmitted behavior highlights the importance of using healthy seeds as propagating material. Finally, the description of the symptoms may help to promptly recognize the disease in crops.

## Figures and Tables

**Table 1:** GenBank accession numbers of DNA sequences of *M. phaseolina* used in phylogenetic analyses.

Species	Isolate	Host	Location	Accession Number		Reference
				ITS	*Tef 1-α*	
*M. phaseolina*	Purdue_SWPAC19-01	*Cucumis sativus* L.	Indiana, USA	MN686333	-	[35]
*M. phaseolina*	Purdue_SWPAC19-01	*Cucumis sativus* L.	Indiana, USA	MN686334	-	[35]
*M. phaseolina*	M1	*Fragaria* × *ananassa*	Italy	MG836711	-	[14]
*M. phaseolina*	CPC 13084	*Phaseolus vulgaris*	Mexico	KF951698.1	KF952070	[11]
*M. phaseolina*	7E64	*Prunus dulcis*	California, USA	KC357271.1	KC357289	[36]
*M. phaseolina*	KWF09	*Actinidia deliciosa*	Turkey	MK287619	-	[37]
*M. phaseolina*	12-K	*Phaseolus vulgaris*	Kyrgyzstan	MW193052.1	-	[38]
*M. phaseolina*	CPC13086	*Solanum melongena*	Mexico	KF951700	-	[11]
*M. phaseolina*	MAC17	*Helianthus annuus*	Italy	GU046807.1	-	Present work
*M. phaseolina*	CBS 205.47	*Phaseolus vulgaris*	Italy	KF951622.1	KF951997	[11]
*M. phaseolina*	CBS 215.35	*Derris elliptica*	Malesya	KF951623.1	-	[11]
*M. phaseolina*	CBS 224.33	*Sesamum indicum*	Uganda	KF951624.1	KF951998	[11]
*M. phaseolina*	CBS 225.33	*Brassica rapa*	Sierra Leone	KF951625	KF951999	[11]
*M. phaseolina*	CBS 226.33	*Nicotiana tabacum*	Palestine	KF951626	-	[11]
*M. phaseolina*	CBS 229.33	*Cajanus indicus*	Sri Lanka	KF951629	KF952002	[11]
*M. phaseolina*	CBS 230.33	*Gossypium herbaceum*	Sudan	KF951630	KF952003	[11]
*M. phaseolina*	CBS 231.33	*Saccharum officinarum*	India	KF951631	KF952004	[11]
*M. phaseolina*	CBS 270.34	*Vigna sinensis*	Missouri, USA	KF951632	KF952005	[11]
*M. phaseolina*	CBS 271.34	*Chrysantemum* sp.	Missouri, USA	KF951633	KF952006	[11]
*M. phaseolina*	CBS 313.51	*Sorghum* sp.	Venezuela	KF951634	KF952007	[11]
*M. phaseolina*	CBS 460.70	*Glycine max*	Denmark	KF951639	-	[11]
*M. phaseolina*	CBS 416.62	*Arachis hypogaea*	Portugal	KF951635	KF952008	[11]
*M. phaseolina*	CBS 457.70	*Phaseolus aureus*	Denmark	KF951636	KF952009	[11]
*M. phaseolina*	CBS 460.70	*Glycine max*	Denmark	KF951639	KF952012	[11]
*M. phaseolina*	CBS 461.70	*Phaseolus vulgaris*	Denmark	KF951640	KF952013	[11]
*M. phaseolina*	CPC 11056	*Vigna unguiculata*	Niger	KF951645	KF952018	[11]
*M. phaseolina*	CPC 11059	*Vigna unguiculata*	Niger	KF951648	KF952021	[11]
*M. phaseolina*	CPC 11062	*Vigna unguiculata*	Niger	KF951651	KF952025	[11]
*M. phaseolina*	CPC 11065	*Vigna unguiculata*	Niger	KF951654	KF952032	[11]
*M. phaseolina*	CPC 11072	*Vigna unguiculata*	Niger	KF951661	KF952035	[11]
*M. phaseolina*	CPC 11076	Soil	Senegal	KF951665	KF952048	[11]
*M. phaseolina*	CPC 11095	Soil	Senegal	KF951678	KF952056	[11]
*M. phaseolina*	CPC 11104	*Panicum miliaceum*	Senegal	KF951686	KF952065	[11]
*M. phaseolina*	CPC 11114	*Panicum miliaceum*	Senegal	KF951693	KF952067	[11]
*M. phaseolina*	CPC 13080	*Sesamum indicum*	Mexico	KF951695	KF952068	[11]
*M. phaseolina*	CPC 13081	*Glycine max*	Mexico	KF951696	KF952070	[11]
*M. phaseolina*	CPC 13082	*Sorghum bicolor*	Mexico	KF951697	KF952073	[11]
*M. phaseolina*	CPC 21387	*Hibiscus sabdarifa*	Senegal	KF951702	KF952076	[11]
*M. phaseolina*	CPC 21392	*Vigna unguiculata*	Senegal	KF951705	KF952080	[11]
*M. phaseolina*	CPC 21405	*Vigna unguiculata*	Senegal	KF951709	KF952120	[11]
*M. phaseolina*	CREA OF 189.2	*Cicer arietinum*	Italy	ON063435	ON181257	Present work
*M. phaseolina*	CREA OF 373.2	*Phaseolus vulgaris*	Italy	ON063434	ON181258	Present work
*B. dothidea*	CMW8000	*Prunus* sp.	Switzerland	AY236949.1	AY236898.1	[11]

## Data Availability

https://www.ncbi.nlm.nih.gov.

## Supplementary Materials

The following supporting information can be downloaded at: https://www.mdpi.com/article/10.3390/pathogens11080842/s1, Figure S1: Morphological characteristics of Macrophomina phaseolina isolates from chickpeas and common beans, in this study. (A) 10 days old colony of Macrophomina phaseolina CREA OF 189.2 and CREA OF 373.2, growing on PDA supplied with antibiotics (A,D). Hyphae of CREA OF 189.2 and CREA OF 373.2 (B,E), with the common bottleneck at the beginning at the right angle hypha. Microsclerotia of CREA OF 189.2 and CREA OF 373.2 (C,F); Figure S2: PCR amplification of calmodulin and tef region by using species-specific primers: Primers MpCal, MsCal and MeCal were used to amplify specifically a ~400 bp fragment from calmodulin gene, while Mptef, Mstef and Metef allowed the differential amplification of ~200 bp fragment from transcription elongation factor; Figure S3: Symptoms of common bean infected with positive control M. phaseolina CREA OF 373.2.
