# Supplementary material for: Occurrence of Macrophomina phaseolina on Chickpea in Italy: Pathogen Identification and Characterization
Source: Pathogens. 2022 Jul 27;11(8):842. doi: 10.3390/pathogens11080842 (PMC9415271; doi:10.3390/pathogens11080842)
Supplement: Supplementary file 1 [file pathogens-11-00842-s001.zip › Macrophomina ITS sequences.pdf]

>MN686333:_Cucumis_sativus_L._Purdue_SWPAC19-01_USA_ITS1
--- --- --- --- --- --- --- --- --- --- --- --- --- --- --- --- --- --- -
-- --- --- --- --- --- --- ---
--- --- --- --- --- --- --- --- --- --- --- --- --T GCT TTG --G CGG GCC
GCG GTC TTC CGC G-- --- --- ---
--G CCG CCC CCC GAT TTT GGG GGG TGG CTA GTG CCC GCC AGA GGA C-- TAT CAA
ACT CCA GTT AGT AAA CGT TGC AGT
CTG -AA AAA AAT ATT AAA TAA ACT AAA ACT TTC AAC AAC GGA TCT CTT GGT TCT
GGC ATC GAT GAA GAA CGC AGC GAA
ATG C-- -GA TAA GTA ATG TGA ATT GCA GAA TTC AGT GAA TCA TCG AAT CTT TGA
ACG CAC ATT GCG CCC CTT GGT ATT
CCG GGG GGC --- --- --- ATG CCT GTT CGA G-- --- -CG TCA TTT CAA CCC TCA
AGC TCT GCT TGG TAT TGG GCA ---
-CC GTC C-T TTG CGG GCG CGC CTC -AA AGA CCT CGG C-G GTG GCG TCT TGC CTC
AAG CGT AGT AGA ATA CAC CTC GCT
TCG GAG CGT AAG GCG TCG CCC GCC GGA CGA ACC TTC TGA AC- TTT TCT CAA GGT
TGA CCT CGG ATC AGG TAG GGA TAC
CCG CTG AAC TTA AGC ATA TCA --- --- ---
>MN686334:_Cucumis_sativus_L._Purdue_SWPAC19-01_USA_ITS4
--- --- --- --- --- --- --- --- --- --- --- --- --A ACC TGC GGA AGG ATC
ATT ACC GAG TTG ATT CGG --G CTC
CGT CCC GAT CCT CCC ACC CTT TGT ATA CCT ACC TCT GTT GCT TTG --G CGG GCC
GCG GTC TTC CGC G-- --- --- ---
--G CCG CCC CCC GAT TTT GGG GGG TGG CTA GTG CCC GCC AGA GGA C-- TAT CAA
ACT CCA GTT AGT AAA CGT TGC AGT
CTG -AA AAA AAT ATT AAA TAA ACT AAA ACT TTC AAC AAC GGA TCT CTT GGT TCT
GGC ATC GAT GAA GAA CGC AGC GAA
ATG C-- -GA TAA GTA ATG TGA ATT GCA GAA TTC AGT GAA TCA TCG AAT CTT TGA
ACG CAC ATT GCG CCC CTT GGT ATT
CCG GGG GGC --- --- --- ATG CCT GTT CGA G-- --- -CG TCA TTT CAA CCC TCA
AGC TCT GCT TGG TAT TGG GCA ---
-CC GTC C-T TTG CGG GCG CGC CTC -AA AGA CCT CGG C-G GTG GCG TCT TGC CTC
AAG CGT AGT AGA ATA CAC CTC GCT
TCG GAG CGT AAG GCG TCG CCC GCC GGA CGA ACC TT- --- --- --- --- --- --- -
-- --- --- --- --- --- --- ---
--- --- --- --- --- --- --- --- --- ---
>MG836711:_Fragaria_x_ananassa_M1_Italy_ITS
--- --- --- --- --- --- --- --- --- --- --- --- --- --- --- --- --- --C
ATT ACC GAG TTG ATT CGG --G CTC
CGT CCC GAT CCT CCC ACC CTT TGT ATA CCT ACC TCT GTT GCT TTG --G CGG GCC
GCG GTC TTC CGC G-- --- --- ---
--G CCG CCC CCC GAT TTT GGG GGG TGG CTA GTG CCC GCC AGA GGA C-- TAT CAA
ACT CCA GTT AGT AAA CGT TGC AGT
CTG -AA AAA AAT ATT AAA TAA ACT AAA ACT TTC AAC AAC GGA TCT CTT GGT TCT
GGC ATC GAT GAA GAA CGC AGC GAA
ATG C-- -GA TAA GTA ATG TGA ATT GCA GAA TTC AGT GAA TCA TCG AAT CTT TGA
ACG CAC ATT GCG CCC CTT GGT ATT
CCG GGG GGC --- --- --- ATG CCT GTT CGA G-- --- -CG TCA TTT CAA CCC TCA
AGC TCT GCT TGG TAT TGG GCA ---
-CC GTC CTT TTG CGG GCG CGC CTC AAA AGA CCT CGG CGG GGG GCG TCT TGC CTC
AAG CGT AGT AGA ATA CAC CTC GCT
TCG GAG CGT AAG GCG TCG CCC GCC GGA CGA ACC TTC TGA ACT TTT TCT CA- --- -
-- --- --- --- --- --- --- ---
--- --- --- --- --- --- --- --- --- ---
>KF951698.1:_(Tassi)_Goidanich_CPC_13084_Mexico_ITS
TAG AGG AAG TAA AAG TCG TAA CAA GGT TTC CGT AGG TGA ACC TGC GGA AGG ATC
ATT ACC GAG TTG ATT CGG --G CTC
CGT CCC GAT CCT CCC ACC CTT TGT ATA CCT ACC TCT GTT GCT TTG --G CGG GCC
GCG GTC TTC CGC G-- --- --- ---

--G CCG CCC CCC GAT TTT GGG GGG TGG CTA GTG CCC GCC AGA GGA C-- TAT CAA ACT CCA GTT AGT AAA CGT TGC AGT
CTG -AA AAA AAT ATT AAA TAA ACT AAA ACT TTC AAC AAC GGA TCT CTT GGT TCT GGC ATC GAT GAA GAA CGC AGC GAA
ATG C-- -GA TAA GTA ATG TGA ATT GCA GAA TTC AGT GAA TCA TCG AAT CTT TGA ACG CAC ATT GCG CCC CTT GGT ATT
CCG GGG GGC --- --- --- ATG CCT GTT CGA G-- --- -CG TCA TTT CAA CCC TCA AGC TCT GCT TGG TAT TGG GCA ---
-CC GTC C-T TTG CGG GCG CGC CTC -AA AGA CCT CGG C-G GTG GCG TCT TGC CTC AAG CGT AGT AGA ATA CAC CTC GCT
TCG GAG CGT AAG GCG TCG CCC GCC GGA CGA ACC TTC TGA AC- TTT TCT CAA GGT TGA CCT CGG ATC AGG TAG GGA TAC
CCG CTG AAC TTA AGC ATA T-- --- --- ---
>KC357271.1:_Prunus_dulcis_7E64_California_USA_ITS
--- --- --- --- --- --- --- --- --- --- --- --- --- --- --- --- -GG ATC ATT ACC GAG TTG ATT CGG --G CTC
CGT CCC GAT CCT CCC ACC CTT TGT ATA CCT ACC TCT GTT GCT TTG --G CGG GCC GCG GTC TTC CGC G-- --- --- ---
--G CCG CCC CCC GAT TTT GGG GGG TGG CTA GTG CCC GCC AGA GGA C-- TAT CAA ACT CCA GTT AGT AAA CGT TGC AGT
CTG -AA AAA AAT ATT AAA TAA ACT AAA ACT TTC AAC AAC GGA TCT CTT GGT TCT GGC ATC GAT GAA GAA CGC AGC GAA
ATG C-- -GA TAA GTA ATG TGA ATT GCA GAA TTC AGT GAA TCA TCG AAT CTT TGA ACG CAC ATT GCG CCC CTT GGT ATT
CCG GGG GGC --- --- --- ATG CCT GTT CGA G-- --- -CG TCA TTT CAA CCC TCA AGC TCT GCT TGG TAT TGG GCA ---
-CC GTC C-T TTG CGG GCG CGC CTC -AA AGA CCT CGG C-G GTG GCG TCT TGC CTC AAG CGT AGT AGA ATA CAC CTC GCT
TCG GAG CGT AAG GCG TCG CCC GCC GGA CGA ACC TTC TGA AC- TTT TCT CAA GGT TGA CCT CGG ATC AGG TAG GGA TAC
CCG CTG AAC TTA AGC ATA TCA AT- --- ---
>MK287619:_Actinidia_chinensis_KWF09_Turkey_ITS
--- --- --- --- --- --- --- --- --- --- --- --- --A ACC TGC GGA AGG ATC ATT ACC GAG TTG ATT CGG --G CTC
CGT CCC GAT CCT CCC ACC CTT TGT ATA CCT ACC TCT GTT GCT TTG --G CGG GCC GCG GTC TTC CGC G-- --- --- ---
--G CCG CCC CCC GAT TTT GGG GGG TGG CTA GTG CCC GCC AGA GGA C-- TAT CAA ACT CCA GTC AGT AAA CGT TGC AGT
CTG -AA AAA AAT ATT AAA TAA ACT AAA ACT TTC AAC AAC GGA TCT CTT GGT TCT GGC ATC GAT GAA GAA CGC AGC GAA
ATG C-- -GA TAA GTA ATG TGA ATT GCA GAA TTC AGT GAA TCA TCG AAT CTT TGA ACG CAC ATT GCG CCC CTT GGT ATT
CCG GGG GGC --- --- --- ATG CCT GTT CGA G-- --- -CG TCA TTT CAA CCC TCA AGC TCT GCT TGG TAT TGG GCA ---
-CC GTC C-T TTG CGG GCG CGC CTC -AA AGA CCT CGG C-G GTG GCG TCT TGC CTC AAG CGT AGT AGA ATA CAC CTC GCT
TCG GAG CGT AAG GCG TCG CCC GCC GGA CGA ACC TTC TGA AC- -TT TCT CAA GG- TGA CCT CGG ATC AGG TAG GGA TA-
--- --- --- --- --- --- --- --- --- ---
>MW193052.1:_Phaseolus_vulgaris_12-K_Kyrgyzstan_ITS
--- GAG TTG ATT CGG --G CTC
CGT CCC GAT CCT CCC ACC CTT TGT ATA CCT ACC TCT GTT GCT TTG --G CGG GCC GCG GTC TTC CGC G-- --- --- ---
--G CCG CCC CCC GAT TTT GGG GGG TGG CTA GTG CCC GCC AGA GGA C-- TAT CAA ACT CCA GTC AGG AAA CGT TGC AGT
CTG AAA AAA AAT ATT AAA TAA ACT AAA ACT TTC AAC AAC GGA TCT CTT GGT TCT GGC ATC GAT GAA GAA CGC AGC GAA
ATG C-- -GA TAA GTA ATG TGA ATT GCA GAA TTC AGT GAA TCA TCG AAT CTT TGA ACG CAC ATT GCG CCC CTT GGT ATT

CCG GGG GGC --- --- --- ATG CCT GTT CGA G-- --- -CG TCA TTT CAA CCC TCA
AGC TCT GCT TGG TAT TGG GCA ---
-CC GTC C-T TTG CGG GCG CGC CTC -AA AGA CCT CGG C-G GTG GCG TCT TGC CTC
AAG CGT AGT AGA ATA CAC CTC GCT
TCG GAG CGT AAG GCG TCG CCC GCC GGA CGA ACC TTC TGA AC- TTT TCT CAA GGT
TGA CCT CGG ATC AGG TAG GGA TAC
CCG CTG AAC TTA AGC ATA TCA ATA AGC GGA
>KF951700:_Solanum_melongena_CPC_13086_Mexico_ITS
TAG AGG AAG TAA AAG TCG TAA CAA GGT TTC CGT AGG TGA ACC TGC GGA AGG ATC
ATT ACC GAG TTG ATT CGG --G CTC
CGT CCC GAT CCT CCC ACC CTT TGT ATA CCT ACC TCT GTT GCT TTG --G CGG GCC
GCG GTC TTC CGC G-- --- --- ---
--G CCG CCC CCC GAT TTT GGG GGG TGG CTA GTG CCC GCC AGA GGA C-- TAT CAA
ACT CCA GTC AGG AAA CGT TGC AGT
CTG AAA AAA AAT ATT AAA TAA ACT AAA ACT TTC AAC AAC GGA TCT CTT GGT TCT
GGC ATC GAT GAA GAA CGC AGC GAA
ATG C-- -GA TAA GTA ATG TGA ATT GCA GAA TTC AGT GAA TCA TCG AAT CTT TGA
ACG CAC ATT GCG CCC CTT GGT ATT
CCG GGG GGC --- --- --- ATG CCT GTT CGA G-- --- -CG TCA TTT CAA CCC TCA
AGC TCT GCT TGG TAT TGG GCA ---
-CC GTC C-T TTG CGG GCG CGC CTC -AA AGA CCT CGG C-G GTG GCG TCT TGC CTC
AAG CGT AGT AGA ATA CAC CTC GCT
TCG GAG CGT AAG GCG TCG CCC GCC GGA CGA ACC TTC TGA AC- TTT TCT CAA GGT
TGA CCT CGG ATC AGG TAG GGA TAC
CCG CTG AAC TTA AGC ATA T-- --- --- ---
>GU046807.1:_Helianthus_annuus_MAC17_Italy_ITS
--- --- --- --- --- --- --- --- --- --- --- --- --- ACC TGC GGA AGG ATC
ATT ACC GAG TTG ATT CGG --G CTC
CGT CCC GAT CCT CCC ACC CTT TGT ATA CCT ACC TCT GTT GCT TTG --G CGG GCC
GCG GTC TTC CGC G-- --- --- ---
--G CCG CCC CCC GAT TTT GGG GGG TGG CTA GTG CCC GCC AGA GGA C-- TAT CAA
ACT CCA GTC AGG AAA CGT TGC AGT
CTG AAA AAA AAT ATT AAA TAA ACT AAA ACT TTC AAC AAC GGA TCT CTT GGT TCT
GGC ATC GAT GAA GAA CGC AGC GAA
ATG C-- -GA TAA GTA ATG TGA ATT GCA GAA TTC AGT GAA TCA TCG AAT CTT TGA
ACG CAC ATT GCG CCC CTT GGT ATT
CCG GGG GGC --- --- --- ATG CCT GTT CGA G-- --- -CG TCA TTT CAA CCC TCA
AGC TCT GCT TGG TAT TGG GCA ---
-CC GTC C-T TTG CGG GCG CGC CTC -AA AGA CCT CGG C-G GTG GCG TCT TGC CTC
AAG CGT AGT AGA ATA CAC CTC GCT
TCG GAG CGT AAG GCG TCG CCC GCC GGA CGA ACC TTC TGA AC- TTT TCT CAA GGT
TGA CCT CGG ATC AGG TAG GGA TAC
CCG CTG AAC TTA AGC ATA T-- --- --- ---
>Cicer_arietinum_CREA_OF_189.2_Italy_ITS1
--- --- --- --- --- --- --- --- --- --- --- --- --- --- --- --- --- --- -
-- --- --- --A GTT CAG AAG GTT
CGT CC- --- --- --- --- --- -GG CGG GCG ACG CCT TAC GCT CCG AAG CGA GGT
GTA TTC TAC TAC GCT TGA GGC AAG
ACG CCA CCG CCG AGG TCT TTG AGG C-- --- GCG CCC G-C AAA GGA CGG TGC CCA
ATA CCA --- AGC AGA GCT TGA GGG
TTG -AA ATG ACG CTC GAA CA- --- --- --- --- --- --- --- --- --- --- ---
GGC AT- --- --- --G CCC CCC GGA
ATA CCA AGG GGC GCA ATG TGC GTT CAA AGA TTC GAT GAT TCA CTG AAT TCT GCA
ATT CAC ATT ACT TAT C-- -GC ATT
TCG CTG CGT TCT TCA TCG ATG CCA GAA CCA AGA GAT CCG TTG TTG AAA GTT TTA
G-- TTT ATT TAA TAT T-- --- ---
--- --- --T TT- --- --- --- TTC --- AGA CTG CAA C-- --- GTT TAC TGA CTG
GAG TTT GAT AG- --- --- TCC TCT
GGC GGG CAC TAG CCA CCC CCC A-- --- --- --- --- --- --- --- --- --- --- -
-- --- -AA ATC GGG GGG CG- ---

--- --- --- --- --- --- --- --- --- ---
>KF951622.1:_Phaseolus_vulgaris_CBS_205.47_Italy_ITS
--- --- --- --- --- --- --- --- --- --- --- --- --- --- --- --A AGG ATC
ATT ACC GAG TTG ATT CGG --G CTC
CGT CCC GAT CCT CCC ACC CTT TGT ATA CCT ACC TCT GTT GCT TTG --G CGG GCC
GCG GTC TTC CGC G-- --- --- ---
--G CCG CCC CCC GAT TTT GGG GGG TGG CTA GTG CCC GCC AGA GGA C-- TAT CAA
ACT CCA GTC AGT AAA CGT TGC AGT
CTG -AA AAA AAT ATT AAA TAA ACT AAA ACT TTC AAC AAC GGA TCT CTT GGT TCT
GGC ATC GAT GAA GAA CGC AGC GAA
ATG C-- -GA TAA GTA ATG TGA ATT GCA GAA TTC AGT GAA TCA TCG AAT CTT TGA
ACG CAC ATT GCG CCC CTT GGT ATT
CCG GGG GGC --- --- --- ATG CCT GTT CGA G-- --- -CG TCA TTT CAA CCC TCA
AGC TCT GCT TGG TAT TGG GCA ---
-CC GTC C-T TTG CGG GCG CGC CTC -AA AGA CCT CGG C-G GTG GCG TCT TGC CTC
AAG CGT AGT AGA ATA CAC CTC GCT
TCG GAG CGT AAG GCG TCG CCC GCC GGA CGA ACC TTC TGA AC- TTT TCT CAA GGT
TGA CCT CGG ATC AGG TAG GGA T--
--- --- --- --- --- --- --- --- --- ---
>KF951623.1:_Derris_elliptica_CBS_215.35_Malesya_ITS
--- --- --- --- --- --- --- --- --- --- --- --- --- --- --- --A AGG ATC
ATT ACC GAG TTG ATT CGG --G CTC
CGT CCC GAT CCT CCC ACC CTT TGT ATA CCT ACC TCT GTT GCT TTG --G CGG GCC
GCG GTC TTC CGC G-- --- --- ---
--G CCG CCC CCC GAT TTT GGG GGG TGG CTA GTG CCC GCC AGA GGA C-- TAT CAA
ACT CCA GTC AGT AAA CGT TGC AGT
CTG -AA AAA AAT ATT AAA TAA ACT AAA ACT TTC AAC AAC GGA TCT CTT GGT TCT
GGC ATC GAT GAA GAA CGC AGC GAA
ATG C-- -GA TAA GTA ATG TGA ATT GCA GAA TTC AGT GAA TCA TCG AAT CTT TGA
ACG CAC ATT GCG CCC CTT GGT ATT
CCG GGG GGC --- --- --- ATG CCT GTT CGA G-- --- -CG TCA TTT CAA CCC TCA
AGC TCT GCT TGG TAT TGG GCA ---
-CC GTC C-T TTG CGG GCG CGC CTC -AA AGA CCT CGG C-G GTG GCG TCT TGC CTC
AAG CGT AGT AGA ATA CAC CTC GCT
TCG GAG CGT AAG GCG TCG CCC GCC GGA CGA ACC TCC TGA AC- TTT TCT CAA GGT
TGA CCT CGG ATC AGG TAG GGA T--
--- --- --- --- --- --- --- --- --- ---
>KF951624.1:_Sesamum_indicum_CBS_224.33_Uganda_ITS
--- --- --- --- --- --- --- --- --- --- --- --- --- --- --- GGA AGG ATC
ATT ACC GAG TTG ATT CGG --G CTC
CGT CCC GAT CCT CCC ACC CTT TGT ATA CCT ACC TCT GTT GCT TTG --G CGG GCC
GCG GTC TTC CGC G-- --- --- ---
--G CCG CCC CCC GAT TTT GGG GGG TGG CTA GTG CCC GCC AGA GGA C-- TAT CAA
ACT CCA GTC AGT AAA CGT TGC AGT
CTG -AA AAA AAT ATT AAA TAA ACT AAA ACT TTC AAC AAC GGA TCT CTT GGT TCT
GGC ATC GAT GAA GAA CGC AGC GAA
ATG C-- -GA TAA GTA ATG TGA ATT GCA GAA TTC AGT GAA TCA TCG AAT CTT TGA
ACG CAC ATT GCG CCC CTT GGT ATT
CCG GGG GGC --- --- --- ATG CCT GTT CGA G-- --- -CG TCA TTT CAA CCC TCA
AGC TCT GCT TGG TAT TGG GCA ---
-CC GTC C-T TTG CGG GCG CGC CTC -AA AGA CCT CGG C-G GTG GCG TCT TGC CTC
AAG CGT AGT AGA ATA CAC CTC GCT
TCG GAG CGT AAG GCG TCG CCC GCC GGA CGA ACC TTC TGA AC- TTT TCT CAA GGT
TGA CCT CGG ATC AGG TAG GGA TA-
--- --- --- --- --- --- --- --- --- ---
>KF951625:_Brassica_rapa_CBS_225.33_Sierra_leone_ITS
--- --- --- --- --- --- --- --- --- --- --- --- --- --- --- --A AGG ATC
ATT ACC GAG TTG ATT CGG --G CTC
CGT CCC GAT CCT CCC ACC CTT TGT ATA CCT ACC TCT GTT GCT TTG --G CGG GCC
GCG GTC TTC CGC G-- --- --- ---

```
--G CCG CCC CCC GAT TTT GGG GGG TGG CTA GTG CCC GCC AGA GGA C-- TAT CAA
ACT CCA GTC AGG AAA CGT TGC AGT
CTG AAA AAA AAT ATT AAA TAA ACT AAA ACT TTC AAC AAC GGA TCT CTT GGT TCT
GGC ATC GAT GAA GAA CGC AGC GAA
ATG C-- -GA TAA GTA ATG TGA ATT GCA GAA TTC AGT GAA TCA TCG AAT CTT TGA
ACG CAC ATT GCG CCC CTT GGT ATT
CCG GGG GGC --- --- --- ATG CCT GTT CGA G-- --- -CG TCA TTT CAA CCC TCA
AGC TCT GCT TGG TAT TGG GCA ---
-CC GTC C-T TTG CGG GCG CGC CTC -AA AGA CCT CGG C-G GTG GCG TCT TGC CTC
AAG CGT AGT AGA ATA CAC CTC GCT
TCG GAG CGT AAG GCG TCG CCC GCC GGA CGA ACC TTC TGA AC- TTT TCT CAA GGT
TGA CCT CGG ATC AGG TAG GGA TA-
--- --- --- --- --- --- --- --- --- ---
>KF951626:_Nicotiana_tabacum_CBS_226.33_Palestine_ITS
--- --- --- --- --- --- --- --- --- --- --- --- --- --- --- --A AGG ATC
ATT ACC GAG TTG ATT CGG --G CTC
CGT CCC GAT CCT CCC ACC CTT TGT ATA CCT ACC TCT GTT GCT TTG --G CGG GCC
GCG GTC TTC CGC G-- --- --- ---
--G CCG CCC CCC GAT TTT GGG GGG TGG CTA GTG CCC GCC AGA GGA C-- TAT CAA
ACT CCA GTC AGT AAA CGT TGC AGT
CTG AAA AAA AAT ATT AAA TAA ACT AAA ACT TTC AAC AAC GGA TCT CTT GGT TCT
GGC ATC GAT GAA GAA CGC AGC GAA
ATG C-- -GA TAA GTA ATG TGA ATT GCA GAA TTC AGT GAA TCA TCG AAT CTT TGA
ACG CAC ATT GCG CCC CTT GGT ATT
CCG GGG GGC --- --- --- ATG CCT GTT CGA G-- --- -CG TCA TTT CAA CCC TCA
AGC TCT GCT TGG TAT TGG GCA ---
-CC GTC C-T TTG CGG GCG CGC CTC -AA AGA CCT CGG C-G GTG GCG TCT TGC CTC
AAG CGT AGT AGA ATA CAC CTC GCT
TCG GAG CGT AAG GCG TCG CCC GCC GGA CGA ACC TTC TGA AC- TTT TCT CAA GGT
TGA CCT CGG ATC AGG TAG GGA TA-
--- --- --- --- --- --- --- --- --- ---
>KF951629:_Cajanus_indicus_CBS_229.33_Sri_Lanka_ITS
--- --- --- --- --- --- --- --- --- --- --- --- --- --- --- --A AGG ATC
ATT ACC GAG TTG ATT CGG --G CTC
CGT CCC GAT CCT CCC ACC CTT TGT ATA CCT ACC TCT GTT GCT TTG --G CGG GCC
GCG GTC TTC CGC G-- --- --- ---
--G CCG CCC CCC GAT TTT GGG GGG TGG CTA GTG CCC GCC AGA GGA C-- TAT CAA
ACT CCA GTC AGT AAA CGT TGC AGT
CTG -AA AAA AAT ATT AAA TAA ACT AAA ACT TTC AAC AAC GGA TCT CTT GGT TCT
GGC ATC GAT GAA GAA CGC AGC GAA
ATG C-- -GA TAA GTA ATG TGA ATT GCA GAA TTC AGT GAA TCA TCG AAT CTT TGA
ACG CAC ATT GCG CCC CTT GGT ATT
CCG GGG GGC --- --- --- ATG CCT GTT CGA G-- --- -CG TCA TTT CAA CCC TCA
AGC TCT GCT TGG TAT TGG GCA ---
-CC GTC C-T TTG CGG GCG CGC CTC -AA AGA CCT CGG C-G GTG GCG TCT TGC CTC
AAG CGT AGT AGA ATA CAC CTC GCT
TCG GAG CGT AAG GCG TCG CCC GCC GGA CGA ACC TTC TGA AC- TTT TCT CAA GGT
TGA CCT CGG ATC AGG TAG GGA TA-
--- --- --- --- --- --- --- --- --- ---
>KF951630:_Gossypium_herbaceum_CBS_230.33_Sudan_ITS
--- --- --- --- --- --- --- --- --- --- --- --- --- --- --- --A AGG ATC
ATT ACC GAG TTG ATT CGG --G CTC
CGT CCC GAT CCT CCC ACC CTT TGT ATA CCT ACC TCT GTT GCT TTG --G CGG GCC
GCG GTC TTC CGC G-- --- --- ---
--G CCG CCC CCC GAT TTT GGG GGG TGG CTA GTG CCC GCC AGA GGA C-- TAT CAA
ACT CCA GTT AGT AAA CGT TGC AGT
CTG -AA AAA AAT ATT AAA TAA ACT AAA ACT TTC AAC AAC GGA TCT CTT GGT TCT
GGC ATC GAT GAA GAA CGC AGC GAA
ATG C-- -GA TAA GTA ATG TGA ATT GCA GAA TTC AGT GAA TCA TCG AAT CTT TGA
ACG CAC ATT GCG CCC CTT GGT ATT
```

CCG GGG GGC --- --- --- ATG CCT GTT CGA G-- --- -CG TCA TTT CAA CCC TCA
AGC TCT GCT TGG TAT TGG GCA ---
-CC GTC C-T TTG CGG GCG CGC CTC -AA AGA CCT CGG C-G GTG GCG TCT TGC CTC
AAG CGT AGT AGA ATA CAC CTC GCT
TCG GAG CGT AAG GCG TCG CCC GCC GGA CGA ACC TTC TGA AC- TTT TCT CAA GGT
TGA CCT CGG ATC AGG TAG GGA TA-
--- --- --- --- --- --- --- --- --- ---
>KF951631:_Saccharum_officinarum_CBS_231.33_India_ITS
TAG AGG AAG TAA AAG TCG TAA CAA GGT TTC CGT AGG TGA ACC TGC GGA AGG ATC
ATT ACC GAG TTG ATT CGG --G CTC
CGT CCC GAT CCT CCC ACC CTT TGT ATA CCT ACC TCT GTT GCT TTG --G CGG GCC
GCG GTC TTC CGC G-- --- --- ---
--G CCG CCC CCC GAT TTT GGG GGG TGG CTA GTG CCC GCC AGA GGA C-- TAT CAA
ACT CCA GTC AGT AAA CGT TGC AGT
CTG -AA AAA AAT ATT AAA TAA ACT AAA ACT TTC AAC AAC GGA TCT CTT GGT TCT
GGC ATC GAT GAA GAA CGC AGC GAA
ATG C-- -GA TAA GTA ATG TGA ATT GCA GAA TTC AGT GAA TCA TCG AAT CTT TGA
ACG CAC ATT GCG CCC CTT GGT ATT
CCG GGG GGC --- --- --- ATG CCT GTT CGA G-- --- -CG TCA TTT CAA CCC TCA
AGC TCT GCT TGG TAT TGG GCA ---
-CC GTC C-T TTG CGG GCG CGC CTC -AA AGA CCT CGG C-G GTG GCG TCT TGC CTC
AAG CGT AGT AGA ATA CAC CTC GCT
TCG GAG CGT AAG GCG TCG CCC GCC GGA CGA ACC TTC TGA AC- TTT TCT CAA GGT
TGA CCT CGG ATC AGG TAG GGA TAC
CCG CTG AAC TTA AGC ATA T-- --- --- ---
>KF951632:_Vigna_sinensis_CBS_270.34_Missouri_USA_ITS
--- --- --- --- --- --- --- --- --- --- --- --- --- --- --- --A AGG ATC
ATT ACC GAG TTG ATT CGG --G CTC
CGT CCC GAT CCT CCC ACC CTT TGT ATA CCT ACC TCT GTT GCT TTG --G CGG GCC
GCG GTC TTC CGC G-- --- --- ---
--G CCG CCC CCC GAT TTT GGG GGG TGG CTA GTG CCC GCC AGA GGA C-- TAT CAA
ACT CCA GTT AGT AAA CGT TGC AGT
CTG -AA AAA AAT ATT AAA TAA ACT AAA ACT TTC AAC AAC GGA TCT CTT GGT TCT
GGC ATC GAT GAA GAA CGC AGC GAA
ATG C-- -GA TAA GTA ATG TGA ATT GCA GAA TTC AGT GAA TCA TCG AAT CTT TGA
ACG CAC ATT GCG CCC CTT GGT ATT
CCG GGG GGC --- --- --- ATG CCT GTT CGA G-- --- -CG TCA TTT CAA CCC TCA
AGC TCT GCT TGG TAT TGG GCA ---
-CC GTC C-T TTG CGG GCG CGC CTC -AA AGA CCT CGG C-G GTG GCG TCT TGC CTC
AAG CGT AGT AGA ATA CAC CTC GCT
TCG GAG CGT AAG GCG TCG CCC GCC GGA CGA ACC TTC TGA AC- TTT TCT CAA GGT
TGA CCT CGG ATC AGG TAG GGA T--
--- --- --- --- --- --- --- --- --- ---
>KF951633:_Chrysanthemum_sp._CBS_271.34_Missouri_USA_ITS
--- --- --- --- --- --- --- --- --- --- --- --- --- --- --- GGA AGG ATC
ATT ACC GAG TTG ATT CGG --G CTC
CGT CCC GAT CCT CCC ACC CTT TGT ATA CCT ACC TCT GTT GCT TTG --G CGG GCC
GCG GTC TTC CGC G-- --- --- ---
--G CCG CCC CCC GAT TTT GGG GGG TGG CTA GTG CCC GCC AGA GGA C-- TAT CAA
ACT CCA GTC AGT AAA CGT TGC AGT
CTG -AA AAA AAT ATT AAA TAA ACT AAA ACT TTC AAC AAC GGA TCT CTT GGT TCT
GGC ATC GAT GAA GAA CGC AGC GAA
ATG C-- -GA TAA GTA ATG TGA ATT GCA GAA TTC AGT GAA TCA TCG AAT CTT TGA
ACG CAC ATT GCG CCC CTT GGT ATT
CCG GGG GGC --- --- --- ATG CCT GTT CGA G-- --- -CG TCA TTT CAA CCC TCA
AGC TCT GCT TGG TAT TGG GCA ---
-CC GTC C-T TTG CGG GCG CGC CTC -AA AGA CCT CGG C-G GTG GCG TCT TGC CTC
AAG CGT AGT AGA ATA CAC CTC GCT
TCG GAG CGT AAG GCG TCG CCC GCC GGA CGA ACC TTC TGA AC- TTT TCT CAA GGT
TGA CCT CGG ATC AGG TAG G-- ---

--- --- --- --- --- --- --- --- --- ---
>KF951634:_Sorghum_sp._CBS_313.51_Venezuela_ITS
TAG AGG AAG TAA AAG TCG TAA CAA GGT TTC CGT AGG TGA ACC TGC GGA AGG ATC
ATT ACC GAG TTG ATT CGG --G CTC
CGT CCC GAT CCT CCC ACC CTT TGT ATA CCT ACC TCT GTT GCT TTG --G CGG GCC
GCG GTC TTC CGC G-- --- --- ---
--G CCG CCC CCC GAT TTT GGG GGT TGG CTA GTG CCC GCC AGA GGA C-- TAT CAA
ACT CCA GTC AGT AAA CGT TGC AGT
CTG -AA AAA AAT ATT AAA TAA ACT AAA ACT TTC AAC AAC GGA TCT CTT GGT TCT
GGC ATC GAT GAA GAA CGC AGC GAA
ATG C-- -GA TAA GTA ATG TGA ATT GCA GAA TTC AGT GAA TCA TCG AAT CTT TGA
ACG CAC ATT GCG CCC CTT GGT ATT
CCG GGG GGC --- --- --- ATG CCT GTT CGA G-- --- -CG TCA TTT CAA CCC TCA
AGC TCT GCT TGG TAT TGG GCA ---
-CC GTC C-T TTG CGG GCG CGC CTC -AA AGA CCT CGG C-G GTG GCG TCT TGC CTC
AAG CGT AGT AGA ATA CAC CTC GCT
TCG GAG CGT AAG GCG TCG CCC GCC GGA CGA ACC TTC TGA AC- TTT TCT CAA GGT
TGA CCT CGG ATC AGG TAG GGA TAC
CCG CTG AAC TT- --- --- --- --- --- ---
>KF951635:_Arachis_hypogea_CBS_416.62_Portugal_ITS
--- --- --- --- --- --- --- --- --- --- --- --- --- --- --- --A AGG ATC
ATT ACC GAG TTG ATT CGG --G CTC
CGT CCC GAT CCT CCC ACC CTT TGT ATA CCT ACC TCT GTT GCT TTG --G CGG GCC
GCG GTC TTC CGC G-- --- --- ---
--G CCG CCC CCC GAT TTT GGG GGG TGG CTA GTG CCC GCC AGA GGA C-- TAT CAA
ACT CCA GTC AGT AAA CGT TGC AGT
CTG -AA AAA AAT ATT AAA TAA ACT AAA ACT TTC AAC AAC GGA TCT CTT GGT TCT
GGC ATC GAT GAA GAA CGC AGC GAA
ATG C-- -GA TAA GTA ATG TGA ATT GCA GAA TTC AGT GAA TCA TCG AAT CTT TGA
ACG CAC ATT GCG CCC CTT GGT ATT
CCG GGG GGC --- --- --- ATG CCT GTT CGA G-- --- -CG TCA TTT CAA CCC TCA
AGC TCT GCT TGG TAT TGG GCA ---
-CC GTC C-T TTG CGG GCG CGC CTC -AA AGA CCT CGG C-G GTG GCG TCT TGC CTC
AAG CGT AGT AGA ATA CAC CTC GCT
TCG GAG CGT AAG GCG TCG CCC GCC GGA CGA ACC TTC TGA AC- TTT TCT CAA GGT
TGA CCT CGG ATC AGG TAG GG- ---
--- --- --- --- --- --- --- --- --- ---
>KF951636:_Phaseolus_aureus_CBS_457.70_Denmark_ITS
TAG AGG AAG TAA AAG TCG TAA CAA GGT TTC CGT AGG TGA ACC TGC GGA AGG ATC
ATT ACC GAG TTG ATT CGG --G CTC
CGT CCC GAT CCT CCC ACC CTT TGT ATA CCT ACC TCT GTT GCT TTG --G CGG GCC
GCG GTC TTC CGC G-- --- --- ---
--G CCG CCC CCC GAT TTT GGG GGG TGG CTA GTG CCC GCC AGA GGA C-- TAT CAA
ACT CCA GTC AGT AAA CGT TGC AGT
CTG -AA AAA AAT ATT AAA TAA ACT AAA ACT TTC AAC AAC GGA TCT CTT GGT TCT
GGC ATC GAT GAA GAA CGC AGC GAA
ATG C-- -GA TAA GTA ATG TGA ATT GCA GAA TTC AGT GAA TCA TCG AAT CTT TGA
ACG CAC ATT GCG CCC CTT GGT ATT
CCG GGG GGC --- --- --- ATG CCT GTT CGA G-- --- -CG TCA TTT CAA CCC TCA
AGC TCT GCT TGG TAT TGG GCA ---
-CC GTC C-T TTG CGG GCG CGC CTC -AA AGA CCT CGG C-G GTG GCG TCT TGC CTC
AAG CGT AGT AGA ATA CAC CTC GCT
TCG GAG CGT AAG GCG TCG CCC GCC GGA CGA ACC TTC TGA AC- TTT TCT CAA GGT
TGA CCT CGG ATC AGG TAG GGA TAC
CC- --- --- --- --- --- --- --- --- ---
>KF951639:_Glycine_max_CBS_460.70_Denmark_ITS
--- --- --- --- --- --- --- --- --- --- --- --- --- --- --- --A AGG ATC
ATT ACC GAG TTG ATT CGG --G CTC
CGT CCC GAT CCT CCC ACC CTT TGT ATA CCT ACC TCT GTT GCT TTG --G CGG GCC
GCG GTC TTC CGC G-- --- --- ---

--G CCG CCC CCC GAT TTT GGG GGG TGG CTA GTG CCC GCC AGA GGA C-- TAT CAA ACT CCA GTC AGT AAA CGT TGC AGT
CTG -AA AAA AAT ATT AAA TAA ACT AAA ACT TTC AAC AAC GGA TCT CTT GGT TCT GGC ATC GAT GAA GAA CGC AGC GAA
ATG C-- -GA TAA GTA ATG TGA ATT GCA GAA TTC AGT GAA TCA TCG AAT CTT TGA ACG CAC ATT GCG CCC CTT GGT ATT
CCG GGG GGC --- --- --- ATG CCT GTT CGA G-- --- -CG TCA TTT CAA CCC TCA AGC TCT GCT TGG TAT TGG GCA ---
-CC GTC C-T TTG CGG GCG CGC CTC -AA AGA CCT CGG C-G GTG GCG TCT TGC CTC AAG CGT AGT AGA ATA CAC CTC GCT
TCG GAG CGT AAG GCG TCG CCC GCC GGA CGA ACC TTC TGA AC- TTT TCT CAA GGT TGA CCT CGG ATC AGG TAG GGA TA-
--- --- --- --- --- --- --- --- --- ---
>KF951640:_Phaseolus_vulgaris_CBS_461.70_Denmark_ITS
TAG AGG AAG TAA AAG TCG TAA CAA GGT TTC CGT AGG TGA ACC TGC GGA AGG ATC ATT ACC GAG TTG ATT CGG --G CTC
CGT CCC GAT CCT CCC ACC CTT TGT ATA CCT ACC TCT GTT GCT TTG --G CGG GCC GCG GTC TTC CGC G-- --- --- ---
--G CCG CCC CCC GAT TTT GGG GGG TGG CTA GTG CCC GCC AGA GGA C-- TAT CAA ACT CCA GTC AGT AAA CGT TGC AGT
CTG -AA AAA AAT ATT AAA TAA ACT AAA ACT TTC AAC AAC GGA TCT CTT GGT TCT GGC ATC GAT GAA GAA CGC AGC GAA
ATG C-- -GA TAA GTA ATG TGA ATT GCA GAA TTC AGT GAA TCA TCG AAT CTT TGA ACG CAC ATT GCG CCC CTT GGT ATT
CCG GGG GGC --- --- --- ATG CCT GTT CGA G-- --- -CG TCA TTT CAA CCC TCA AGC TCT GCT TGG TAT TGG GCA ---
-CC GTC C-T TTG CGG GCG CGC CTC -AA AGA CCT CGG C-G GTG GCG TCT TGC CTC AAG CGT AGT AGA ATA CAC CTC GCT
TCG GAG CGT AAG GCG TCG CCC GCC GGA CGA ACC TTC TGA AC- TTT TCT CAA GGT TGA CCT CGG ATC AGG TAG G-- ---
--- --- --- --- --- --- --- --- --- ---
>KF951645:_Vigna_unguiculata_CPC_11056_Niger_ITS
--- --- --- --- --- --- --- --- --- --- --- --- --- --- --- GGA AGG ATC ATT ACC GAG TTG ATT CGG --G CTC
CGT CCC GAT CCT CCC ACC CTT TGT ATA CCT ACC TCT GTT GCT TTG --G CGG GCC GCG GTC TTC CGC G-- --- --- ---
--G CCG CCC CCC GAT TTT GGG GGG TGG CTA GTG CCC GCC AGA GGA C-- TAT CAA ACT CCA GTC AGT AAA CGT TGC AGT
CTG -AA AAA AAT ATT AAA TAA ACT AAA ACT TTC AAC AAC GGA TCT CTT GGT TCT GGC ATC GAT GAA GAA CGC AGC GAA
ATG C-- -GA TAA GTA ATG TGA ATT GCA GAA TTC AGT GAA TCA TCG AAT CTT TGA ACG CAC ATT GCG CCC CTT GGT ATT
CCG GGG GGC --- --- --- ATG CCT GTT CGA G-- --- -CG TCA TTT CAA CCC TCA AGC TCT GCT TGG TAT TGG GCA ---
-CC GTC C-T TTG CGG GCG CGC CTC -AA AGA CCT CGG C-G GTG GCG TCT TGC CTC AAG CGT AGT AGA ATA CAC CTC GCT
TCG GAG CGT AAG GCG TCG CCC GCC GGA CGA ACC TTC TGA AC- TTT TCT CAA GGT TGA CCT CGG ATC AGG T-- --- ---
--- --- --- --- --- --- --- --- --- ---
>KF951648:_Vigna_unguiculata_CPC_11059_Niger_ITS
--- --- --- --- --- --- --- --- --- --- --- --- --- --- --- GGA AGG ATC ATT ACC GAG TTG ATT CGG --G CTC
CGT CCC GAT CCT CCC ACC CTT TGT ATA CCT ACC TCT GTT GCT TTG --G CGG GCC GCG GTC TTC CGC G-- --- --- ---
--G CCG CCC CCC GAT TTT GGG GGG TGG CTA GTG CCC GCC AGA GGA C-- TAT CAA ACT CCA GTC AGT AAA CGT TGC AGT
CTG -AA AAA AAT ATT AAA TAA ACT AAA ACT TTC AAC AAC GGA TCT CTT GGT TCT GGC ATC GAT GAA GAA CGC AGC GAA
ATG C-- -GA TAA GTA ATG TGA ATT GCA GAA TTC AGT GAA TCA TCG AAT CTT TGA ACG CAC ATT GCG CCC CTT GGT ATT

CCG GGG GGC --- --- --- ATG CCT GTT CGA G-- --- -CG TCA TTT CAA CCC TCA
AGC TCT GCT TGG TAT TGG GCA ---
-CC GTC C-T TTG CGG GCG CGC CTC -AA AGA CCT CGG C-G GTG GCG TCT TGC CTC
AAG CGT AGT AGA ATA CAC CTC GCT
TCG GAG CGT AAG GCG TCG CCC GCC GGA CGA ACC TTC TGA AC- TTT TCT CAA GGT
TGA CCT CGG ATC AGG T-- --- ---
--- --- --- --- --- --- --- --- --- ---
>KF951651:_Vigna_unguiculata_CPC_11062_Niger_ITS
--- --- --- --- --- --- --- --- --- --- --- --- --- --- --- GGA AGG ATC
ATT ACC GAG TTG ATT CGG --G CTC
CGT CCC GAT CCT CCC ACC CTT TGT ATA CCT ACC TCT GTT GCT TTG --G CGG GCC
GCG GTC TTC CGC G-- --- --- ---
--G CCG CCC CCC GAT TTT GGG GGG TGG CTA GTG CCC GCC AGA GGA C-- TAT CAA
ACT CCA GTC AGT AAA CGT TGC AGT
CTG -AA AAA AAT ATT AAA TAA ACT AAA ACT TTC AAC AAC GGA TCT CTT GGT TCT
GGC ATC GAT GAA GAA CGC AGC GAA
ATG C-- -GA TAA GTA ATG TGA ATT GCA GAA TTC AGT GAA TCA TCG AAT CTT TGA
ACG CAC ATT GCG CCC CTT GGT ATT
CCG GGG GGC --- --- --- ATG CCT GTT CGA G-- --- -CG TCA TTT CAA CCC TCA
AGC TCT GCT TGG TAT TGG GCA ---
-CC GTC C-T TTG CGG GCG CGC CTC -AA AGA CCT CGG C-G GTG GCG TCT TGC CTC
AAG CGT AGT AGA ATA CAC CTC GCT
TCG GAG CGT AAG GCG TCG CCC GCC GGA CGA ACC TTC TGA AC- TTT TCT CAA GGT
TGA CCT CGG ATC AGG T-- --- ---
--- --- --- --- --- --- --- --- --- ---
>KF951654:_Vigna_unguiculata_CPC_11065_Niger_ITS
--- --- --- --- --- --- --- --- --- --- --- --- --- --- --- GGA AGG ATC
ATT ACC GAG TTG ATT CGG --G CTC
CGT CCC GAT CCT CCC ACC CTT TGT ATA CCT ACC TCT GTT GCT TTG --G CGG GCC
GCG GTC TTC CGC G-- --- --- ---
--G CCG CCC CCC GAT TTT GGG GGG TGG CTA GTG CCC GCC AGA GGA C-- TAT CAA
ACT CCA GTC AGT AAA CGT TGC AGT
CTG -AA AAA AAT ATT AAA CAA ACT AAA ACT TTC AAC AAC GGA TCT CTT GGT TCT
GGC ATC GAT GAA GAA CGC AGC GAA
ATG C-- -GA TAA GTA ATG TGA ATT GCA GAA TTC AGT GAA TCA TCG AAT CTT TGA
ACG CAC ATT GCG CCC CTT GGT ATT
CCG GGG GGC --- --- --- ATG CCT GTT CGA G-- --- -CG TCA TTT CAA CCC TCA
AGC TCT GCT TGG TAT TGG GCA ---
-CC GTC C-T TTG CGG GCG CGC CTC -AA AGA CCT CGG C-G GTG GCG TCT TGC CTC
AAG CGT AGT AGA ATA CAC CTC GCT
TCG GAG CGT AAG GCG TCG CCC GCC GGA CGA ACC TTC TGA AC- TTT TCT CAA GGT
TGA CCT CGG ATC AGG T-- --- ---
--- --- --- --- --- --- --- --- --- ---
>KF951661:_Vigna_unguiculata_CPC_11072_Niger_ITS
--- --- --- --- --- --- --- --- --- --- --- --- --- --- --- GGA AGG ATC
ATT ACC GAG TTG ATT CGG --G CTC
CGT CCC GAT CCT CCC ACC CTT TGT ATA CCT ACC TCT GTT GCT TTG --G CGG GCC
GCG GTC TTC CGC G-- --- --- ---
--G CCG CCC CCC GAT TTT GGG GGG TGG CTA GTG CCC GCC AGA GGA C-- TAT CAA
ACT CCA GTC AGT AAA CGT TGC AGT
CTG -AA AAA AAT ATT AAA TAA ACT AAA ACT TTC AAC AAC GGA TCT CTT GGT TCT
GGC ATC GAT GAA GAA CGC AGC GAA
ATG C-- -GA TAA GTA ATG TGA ATT GCA GAA TTC AGT GAA TCA TCG AAT CTT TGA
ACG CAC ATT GCG CCC CTT GGT ATT
CCG GGG GGC --- --- --- ATG CCT GTT CGA G-- --- -CG TCA TTT CAA CCC TCA
AGC TCT GCT TGG TAT TGG GCA ---
-CC GTC C-T TTG CGG GCG CGC CTC -AA AGA CCT CGG C-G GTG GCG TCT TGC CTC
AAG CGT AGT AGA ATA CAC CTC GCT
TCG GAG CGT AAG GCG TCG CCC GCC GGA CGA ACC TTC TGA AC- TTT TCT CAA GGT
TGA CCT CGG ATC AGG TAG G-- ---

--- --- --- --- --- --- --- --- --- ---
>KF951665:_Soil_CPC_11076_Senegal_ITS
--- --- --- --- --- --- --- --- --- --- --- --- --- --- --- GGA AGG ATC
ATT ACC GAG TTG ATT CGG --G CTC
CGT CCC GAT CCT CCC ACC CTT TGT ATA CCT ACC TCT GTT GCT TTG --G CGG GCC
GCG GTC TTC CGC G-- --- --- ---
--G CCG CCC CCC GAT TTT GGG GGG TGG CTA GTG CCC GCC AGA GGA C-- TAT CAA
ACT CCA GTC AGT AAA CGT TGC AGT
CTG -AA AAA AAT ATT AAA TAA ACT AAA ACT TTC AAC AAC GGA TCT CTT GGT TCT
GGC ATC GAT GAA GAA CGC AGC GAA
ATG C-- -GA TAA GTA ATG TGA ATT GCA GAA TTC AGT GAA TCA TCG AAT CTT TGA
ACG CAC ATT GCG CCC CTT GGT ATT
CCG GGG GGC --- --- --- ATG CCT GTT CGA G-- --- -CG TCA TTT CAA CCC TCA
AGC TCT GCT TGG TAT TGG GCA ---
-CC GTC C-T TTG CGG GCG CGC CTC -AA AGA CCT CGG C-G GTG GCG TCT TGC CTC
AAG CGT AGT AGA ATA CAC CTC GCT
TCG GAG CGT AAG GCG TCG CCC GCC GGA CGA ACC TTC TGA AC- TTT TCT CAA GGT
TGA CCT CGG ATC AGG T-- --- ---
--- --- --- --- --- --- --- --- --- ---
>KF951678:_Soil_CPC_11095_Senegal_ITS
TAG AGG AAG TAA AAG TCG TAA CAA GGT TTC CGT AGG TGA ACC TGC GGA AGG ATC
ATT ACC GAG TTG ATT CGG --G CTC
CGT CCC GAT CCT CCC ACC CTT TGT ATA CCT ACC TCT GTT GCT TTG --G CGG GCC
GCG GTC TTC CGC G-- --- --- ---
--G CCG CCC CCC GAT TTT GGG GGG TGG CTA GTG CCC GCC AGA GGA C-- TAT CAA
ACT CCA GTC AGT AAA CGT TGC AGT
CTG -AA AAA AAT ATT AAA TAA ACT AAA ACT TTC AAC AAC GGA TCT CTT GGT TCT
GGC ATC GAT GAA GAA CGC AGC GAA
ATG C-- -GA TAA GTA ATG TGA ATT GCA GAA TTC AGT GAA TCA TCG AAT CTT TGA
ACG CAC ATT GCG CCC CTT GGT ATT
CCG GGG GGC --- --- --- ATG CCT GTT CGA G-- --- -CG TCA TTT CAA CCC TCA
AGC TCT GCT TGG TAT TGG GCA CCG
TCC GTC C-T TTG CGG GCG CGC CTC -AA AGA CCT CGG C-G GTG GCG TCT TGC CTC
AAG CGT AGT AGA ATA CAC CTC GCT
TCG GAG CGT AAG GCG TCG CCC GCC GGA CGA ACC TTC TGA AC- TTT TCT CAA GGT
TGA CCT CGG ATC AGG TAG GGA T--
--- --- --- --- --- --- --- --- --- ---
>KF951686:_Panicum_miliaceum_CPC_11104_Senegal_ITS
--- --- --- --- --- --- --- --- --- --- --- --- --- --- --- --A AGG ATC
ATT ACC GAG TTG ATT CGG --G CTC
CGT CCC GAT CCT CCC ACC CTT TGT ATA CCT ACC TCT GTT GCT TTG --G CGG GCC
GCG GTC TTC CGC G-- --- --- ---
--G CCG CCC CCC GAT TTT GGG GGG TGG CTA GTG CCC GCC AGA GGA C-- TAT CAA
ACT CCA GTC AGT AAA CGT TGC AGT
CTG -AA AAA AAT ATT AAA TAA ACT AAA ACT TTC AAC AAC GGA TCT CTT GGT TCT
GGC ATC GAT GAA GAA CGC AGC GAA
ATG C-- -GA TAA GTA ATG TGA ATT GCA GAA TTC AGT GAA TCA TCG AAT CTT TGA
ACG CAC ATT GCG CCC CTT GGT ATT
CCG GGG GGC --- --- --- ATG CCT GTT CGA G-- --- -CG TCA TTT CAA CCC TCA
AGC TCT GCT TGG TAT TGG GCA ---
-CC GTC C-T TTG CGG GCG CGC CTC -AA AGA CCT CGG C-G GTG GCG TCT TGC CTC
AAG CGT AGT AGA ATA CAC CTC GCT
TCG GAG CGT AAG GCG TCG CCC GCC GGA CGA ACC TTC TGA AC- TTT TCT CAA GGT
TGA CCT CGG ATC AGG TAG GGA T--
--- --- --- --- --- --- --- --- --- ---
>KF951693:Panicum_miliaceum_CPC_11114_Senegal_ITS
TAG AGG AAG TAA AAG TCG TAA CAA GGT TTC CGT AGG TGA ACC TGC GGA AGG ATC
ATT ACC GAG TTG ATT CGG --G CTC
CGT CCC GAT CCT CCC ACC CTT TGT ATA CCT ACC TCT GTT GCT TTG --G CGG GCC
GCG GTC TTC CGC G-- --- --- ---

--G CCG CCC CCC GAT TTT GGG GGG TGG CTA GTG CCC GCC AGA GGA C-- TAT CAA
ACT CCA GTC AGT AAA CGT TGC AGT
CTG -AA AAA AAT ATT AAA TAA ACT AAA ACT TTC AAC AAC GGA TCT CTT GGT TCT
GGC ATC GAT GAA GAA CGC AGC GAA
ATG C-- -GA TAA GTA ATG TGA ATT GCA GAA TTC AGT GAA TCA TCG AAT CTT TGA
ACG CAC ATT GCG CCC CTT GGT ATT
CCG GGG GGC --- --- --- ATG CCT GTT CGA G-- --- -CG TCA TTT CAA CCC TCA
AGC TCT GCT TGG TAT TGG GCA ---
-CC GTC C-T TTG CGG GCG CGC CTC -AA AGA CCT CGG C-G GTG GCG TCT TGC CTC
AAG CGT AGT AGA ATA CAC CTC GCT
TCG GAG CGT AAG GCG TCG CCC GCC GGA CGA ACC TTC TGA AC- TTT TCT CAA GGT
TGA CCT CGG ATC AGG TAG GGA T--
--- --- --- --- --- --- --- --- --- ---
>KF951695:_Sesamum_indicum_CPC_13080_Mexico_ITS
TAG AGG AAG TAA AAG TCG TAA CAA GGT TTC CGT AGG TGA ACC TGC GGA AGG ATC
ATT ACC GAG TTG ATT CGG --G CTC
CGT CCC GAT CCT CCC ACC CTT TGT ATA CCT ACC TCT GTT GCT TTG --G CGG GCC
GCG GTC TTC CGC G-- --- --- ---
--G CCG CCC CCC GAT TTT GGG GGG TGG CTA GTG CCC GCC AGA GGA C-- TAT CAA
ACT CCA GTC AGT AAA CGT TGC AGT
CTG -AA AAA AAT ATT AAA TAA ACT AAA ACT TTC AAC AAC GGA TCT CTT GGT TCT
GGC ATC GAT GAA GAA CGC AGC GAA
ATG C-- -GA TAA GTA ATG TGA ATT GCA GAA TTC AGT GAA TCA TCG AAT CTT TGA
ACG CAC ATT GCG CCC CTT GGT ATT
CCG GGG GGC --- --- --- ATG CCT GTT CGA G-- --- -CG TCA TTT CAA CCC TCA
AGC TCT GCT TGG TAT TGG GCA ---
-CC GTC C-T TTG CGG GCG CGC CTC -AA AGA CCT CGG C-G GTG GCG TCT TGC CTC
AAG CGT AGT AGA ATA CAC CTC GCT
TCG GAG CGT AAG GCG TCG CCC GCC GGA CGA ACC TTC TGA AC- TTT TCT CAA GGT
TGA CCT CGG ATC AGG TAG GGA TAC
CCG CTG AAC TTA AGC ATA T-- --- --- ---
>KF951696:_Glycine_max_CPC_13081_Mexico_ITS
TAG AGG AAG TAA AAG TCG TAA CAA GGT TTC CGT AGG TGA ACC TGC GGA AGG ATC
ATT ACC GAG TTG ATT CGG --G CTC
CGT CCC GAT CCT CCC ACC CTT TGT ATA CCT ACC TCT GTT GCT TTG --G CGG GCC
GCG GTC TTC CGC G-- --- --- ---
--G CCG CCC CCC GAT TTT GGG GGG TGG CTA GTG CCC GCC AGA GGA C-- TAT CAA
ACT CCA GTC AGT AAA CGT TGC AGT
CTG -AA AAA AAT ATT AAA TAA ACT AAA ACT TTC AAC AAC GGA TCT CTT GGT TCT
GGC ATC GAT GAA GAA CGC AGC GAA
ATG C-- -GA TAA GTA ATG TGA ATT GCA GAA TTC AGT GAA TCA TCG AAT CTT TGA
ACG CAC ATT GCG CCC CTT GGT ATT
CCG GGG GGC --- --- --- ATG CCT GTT CGA G-- --- -CG TCA TTT CAA CCC TCA
AGC TCT GCT TGG TAT TGG GCA ---
-CC GTC C-T TTG CGG GCG CGC CTC -AA AGA CCT CGG C-G GTG GCG TCT TGC CTC
AAG CGT AGT AGA ATA CAC CTC GCT
TCG GAG CGT AAG GCG TCG CCC GCC GGA CGA ACC TTC TGA AC- TTT TCT CAA GGT
TGA CCT CGG ATC AGG TAG GGA TAC
CCG CTG AAC TTA AGC ATA T-- --- --- ---
>KF951697:_Sorghum_bicolor_CPC_13082_Mexico_ITS
TAG AGG AAG TAA AAG TCG TAA CAA GGT TTC CGT AGG TGA ACC TGC GGA AGG ATC
ATT ACC GAG TTG ATT CGG --G CTC
CGT CCC GAT CCT CCC ACC CTT TGT ATA CCT ACC TCT GTT GCT TTG --G CGG GCC
GCG GTC TTC CGC G-- --- --- ---
--G CCG CCC CCC GAT TTT GGG GGG TGG CTA GTG CCC GCC AGA GGA C-- TAT CAA
ACT CCA GTC AGT AAA CGT TGC AGT
CTG -AA AAA AAT ATT AAA TAA ACT AAA ACT TTC AAC AAC GGA TCT CTT GGT TCT
GGC ATC GAT GAA GAA CGC AGC GAA
ATG C-- -GA TAA GTA ATG TGA ATT GCA GAA TTC AGT GAA TCA TCG AAT CTT TGA
ACG CAC ATT GCG CCC CTT GGT ATT

CCG GGG GGC --- --- --- ATG CCT GTT CGA G-- --- -CG TCA TTT CAA CCC TCA
AGC TCT GCT TGG TAT TGG GCA ---
-CC GTC C-T TTG CGG GCG CGC CTC -AA AGA CCT CGG C-G GTG GCG TCT TGC CTC
AAG CGT AGT AGA ATA CAC CTC GCT
TCG GAG CGT AAG GCG TCG CCC GCC GGA CGA ACC TTC TGA AC- TTT TCT CAA GGT
TGA CCT CGG ATC AGG TA- --- ---
--- --- --- --- --- --- --- --- --- ---
>KF951702:_Hibiscus_sabdarifa_CPC_21387_Senegal_ITS
--- --- --- --- --- --- --- --- --- --- --- --- --- --- -GC GGA AGG ATC
ATT ACC GAG TTG ATT CGG --G CTC
CGT CCC GAT CCT CCC ACC CTT TGT ATA CCT ACC TCT GTT GCT TTG --G CGG GCC
GCG GTC TTC CGC G-- --- --- ---
--G CCG CCC CCC GAT TTT GGG GGG TGG CTA GTG CCC GCC AGA GGA C-- TAT CAA
ACT CCA GTC AGT AAA CGT TGC AGT
CTG -AA AAA AAT ATT AAA TAA ACT AAA ACT TTC AAC AAC GGA TCT CTT GGT TCT
GGC ATC GAT GAA GAA CGC AGC GAA
ATG C-- -GA TAA GTA ATG TGA ATT GCA GAA TTC AGT GAA TCA TCG AAT CTT TGA
ACG CAC ATT GCG CCC CTT GGT ATT
CCG GGG GGC --- --- --- ATG CCT GTT CGA G-- --- -CG TCA TTT CAA CCC TCA
AGC TCT GCT TGG TAT TGG GCA CCG
TCC GTC C-T TTG CGG GCG CGC CTC -AA AGA CCT CGG C-G GTG GCG TCT TGC CTC
AAG CGT AGT AGA ATA CAC CTC GCT
TCG GAG CGT AAG GCG TCG CCC GCC GGA CGA ACC TTC TGA AC- TTT TCT CAA GGT
TGA CCT CGG ATC AGG TAG GGA TAC
CCG CTG AAC TTA AGC ATA T-- --- --- ---
>KF951705:_Vigna_unguiculata_CPC_21392_Senegal_ITS
--- --- --- --- --- --- --- --- --- --- --- --- --- --- --- --- --- --- -
TT ACC GAG TTG ATT CGG --G CTC
CGT CCC GAT CCT CCC ACC CTT TGT ATA CCT ACC TCT GTT GCT TTG --G CGG GCC
GCG GTC TTC CGC G-- --- --- ---
--G CCG CCC CCC GAT TTT GGG GGG TGG CTA GTG CCC GCC AGA GGA C-- TAT CAA
ACT CCA GTC AGT AAA CGT TGC AGT
CTG -AA AAA AAT ATT AAA TAA ACT AAA ACT TTC AAC AAC GGA TCT CTT GGT TCT
GGC ATC GAT GAA GAA CGC AGC GAA
ATG C-- -GA TAA GTA ATG TGA ATT GCA GAA TTC AGT GAA TCA TCG AAT CTT TGA
ACG CAC ATT GCG CCC CTT GGT ATT
CCG GGG GGC --- --- --- ATG CCT GTT CGA G-- --- -CG TCA TTT CAA CCC TCA
AGC TCT GCT TGG TAT TGG GCA CCG
TCC GTC C-T TTG CGG GCG CGC CTC -AA AGA CCT CGG C-G GTG GCG TCT TGC CTC
AAG CGT AGT AGA ATA CAC CTC GCT
TCG GAG CGT AAG GCG TCG CCC GCC GGA CGA ACC TTC TGA AC- TTT TCT CAA GGT
TGA CCT CGG ATC AGG TAG GGA TAC
CCG CTG AAC TTA AGC ATA T-- --- --- ---
>KF951709:_Vigna_unguiculata_cPC_21405_Senegal_ITS
--- --- --- --- --- --- --- --- --- --- --- --- --- --- --- --- --- --- -
TT ACC GAG TTG ATT CGG --G CTC
CGT CCC GAT CCT CCC ACC CTT TGT ATA CCT ACC TCT GTT GCT TTG --G CGG GCC
GCG GTC TTC CGC G-- --- --- ---
--G CCG CCC CCC GAT TTT GGG GGG TGG CTA GTG CCC GCC AGA GGA C-- TAT CAA
ACT CCA GTC AGT AAA CGT TGC AGT
CTG -AA AAA AAT ATT AAA TAA ACT AAA ACT TTC AAC AAC GGA TCT CTT GGT TCT
GGC ATC GAT GAA GAA CGC AGC GAA
ATG C-- -GA TAA GTA ATG TGA ATT GCA GAA TTC AGT GAA TCA TCG AAT CTT TGA
ACG CAC ATT GCG CCC CTT GGT ATT
CCG GGG GGC --- --- --- ATG CCT GTT CGA G-- --- -CG TCA TTT CAA CCC TCA
AGC TCT GCT TGG TAT TGG GCA ---
-CC GTC C-T TTG CGG GCG CGC CTC -AA AGA CCT CGG C-G GTG GCG TCT TGC CTC
AAG CGT AGT AGA ATA CAC CTC GCT
TCG GAG CGT AAG GCG TCG CCC GCC GGA CGA ACC TTC TGA AC- TTT TCT CAA GGT
TGA CCT CGG ATC AGG TAG GGA TAC

CCG CTG AAC TTA AGC ATA T-- --- --- ---
>KF951752:_Abelmoschus_esculentus_CPC_21470_Senegal_ITS
--- --- --- --- --- --- --- --- --- --- --- --- --- --- --- --- --- --C
ATT ACC GAG TTG ATT CGG --G CTC
CGT CCC GAT CCT CCC ACC CTT TGT ATA CCT ACC TCT GTT GCT TTG --G CGG GCC
GCG GTC TTC CGC G-- --- --- ---
--G CCG CCC CCC GAT TTT GGG GGG TGG CTA GTG CCC GCC AGA GGA C-- TAT CAA
ACT CCA GTC AGT AAA CGT TGC AGT
CTG -AA AAA AAT ATT AAA TAA ACT AAA ACT TTC AAC AAC GGA TCT CTT GGT TCT
GGC ATC GAT GAA GAA CGC AGC GAA
ATG C-- -GA TAA GTA ATG TGA ATT GCA GAA TTC AGT GAA TCA TCG AAT CTT TGA
ACG CAC ATT GCG CCC CTT GGT ATT
CCG GGG GGC --- --- --- ATG CCT GTT CGA G-- --- -CG TCA TTT CAA CCC TCA
AGC TCT GCT TGG TAT TGG GCA ---
-CC GTC C-T TTG CGG GCG CGC CTC -AA AGA CCT CGG C-G GTG GCG TCT TGC CTC
AAG CGT AGT AGA ATA CAC CTC GCT
TCG GAG CGT AAG GCG TCG CCC GCC GGA CGA ACC TTC TGA AC- TTT TCT CAA GGT
TGA CCT CGG ATC AGG TAG GGA TAC
CCG CTG AAC TTA AGC ATA T-- --- --- ---
>Phaseoulus_vulgaris_CREA_OF_373.2_Italy_ITS
--- --- --- --- --- --- --- --- --- --- --- --- --- --- --- -CC GAG GTC
AAC CTT GAG AAA GTT CAG AAG GTT
CGT CC- --- --- --- --- --- -GG CGG GCG ACG CCT TAC GCT CCG AAG CGA GGT
GTA TTC TAC TAC GCT TGA GGC AAG
ACG CCA CCG CCG AGG TCT TTG AGG C-- --- GCG CCC G-C AAA GGA CGG TGC CCA
ATA CCA --- AGC AGA GCT TGA GGG
TTG -AA ATG ACG CTC GAA CA- --- --- --- --- --- --- --- --- --- --- ---
GGC AT- --- --- --G CCC CCC GGA
ATA CCA AGG GGC GCA ATG TGC GTT CAA AGA TTC GAT GAT TCA CTG AAT TCT GCA
ATT CAC ATT ACT TAT C-- -GC ATT
TCG CTG CGT TCT TCA TCG ATG CCA GAA CCA AGA GAT CCG TTG TTG AAA GTT TTA
G-- TTT ATT TAA TAT T-- --- ---
--- --- --T TT- --- --- --- TTC --- AGA CTG CAA C-- --- GTT TAC TGA CTG
GAG TTT GAT AG- --- --- TCC TCT
GGC GGG CAC TAG CCA CCC CCC A-- --- --- --- --- --- --- --- --- --- --- -
-- --- -AA ATC GGG GGG CGG ---
--- --- --- --- --- --- --- --- --- ---
