# Supplementary material for: Occurrence of Macrophomina phaseolina on Chickpea in Italy: Pathogen Identification and Characterization
Source: Pathogens. 2022 Jul 27;11(8):842. doi: 10.3390/pathogens11080842 (PMC9415271; doi:10.3390/pathogens11080842)
Supplement: Supplementary file 1 [file pathogens-11-00842-s001.zip › Macrophomina TEF sequences.pdf]

>KF952070:_Phaseolus_vulgaris_CPC_13084_Mexico_TEF
--G AGA AGG TAA ACA CAC TTT TCG CAC TCC TGC ACA TGA GGC TGT CGC TGC ACC
AGA ATT TGC CGT ATC TCA TTG ACT
GAG GGG CA- TTT TTT GGT GGG GTT GGT CCG CGC TAA GCC GCA TTC AGC CTT CGG
CAA AAT CTC CGC ATG TGG A-- TTT
TTT TTT --G ACC GGC GTG CGA CCG ATG CGC GAG CCC CTC CAC ATT TTT GCC ACT
CGT GTT CTT CTC TGC GAG CAT AAG
CTA ACT GCG GCT ATT ATA GGA AGC CGC TGA GCT CGG --- ---
>KC357289:_Prunus_dulcis_7E64_California_USA_TEF
TCG AGA AGG TAA ACA CAC TTT TCG CAC TCC TGC ACA TGA GGC TGT CGC TGC ACC
AGA ATT TGC CGT ATC TCA TTG ACT
GAG GGG CA- TTT TTT GGT GGG GTT GGT CCG CGC TAA GCC GCA TTC AGC CTT CGG
CAA AAT CTC CGC ATG TGG A-- TTT
TTT TTT --G ACC GGC GTG CGA CCG ATG CGC GAG CCC CTC CAC ATT TTT GCC ACT
CGT GTT CTT CTC TGC GAG CAT AAG
CTA ACT GCG GCT ATT ATA GGA AGC CGC TGA GCT CGG TAA GGG
>CREA_OF_189.2_Cicer_arietinum_CREA_OF_189_Italy_TEF
--- --- --- --- --- --- --- --- --- --- --- --- --- -GC TGT CGC TGC ACC
AGA ATT TGC CGT ATC TCA TTG ACT
GAG GGG CA- TTT TTT GGT GGG GTT GGT CCG CGC TAA GCC GCA TTC AGC CCT CGG
CAA AAT CTC CGC ATG TGG A-- -TT
TTT TTT TCG ACC GGC GTG CGA CCG ATG CGC GAG CCC CTC CAC ATT TTT GCC ACT
CGT GTT CTT CTC TGC GAG CAT AAG
CTA ACT GCG GCT ATT ATA GGA AGC CGC TGA GCT CGG TAA GG-
>KF951997:_Phaseolus_vulgaris_CBS_205.47_Italy_TEF
--G AGA AGG TAA ACA CAC TTT TCG CAC TCC TGC GCA TGA GGC TGT CGC TGC ACC
AGA ATT TGC CGT ACC TCA TTG ACT
GAG GGG CA- TTT TTT GGT GGG GTT GGT CCG CGC TAA GCC GCA TTC AGC CTT CGG
CAA AAT CTC CGC ATG TGG A-- -TT
TTT TTT TCG GCC GGC GTG CGA CCG ATG CGC GAG CCC CTC CAC ATT TTT GCC ACT
CGT GTT CTT CTC TGC GAG CAT AAG
CTA ACT GCG GCT ATT ATA GGA AGC CGC TGA GCT CGG --- ---
>KF951998:_Sesamum_indicum_CBS_224.33_Uganda_TEF
--G AGA AGG TAA ACA CAC TTT TCG CAC TCC TGC ACA TGA GGC TGT CGC TGC ACC
AGA ATT TGC CGT ACC TCA TTG ACT
GAG GGG CA- -TT TTT GGT GGG GTT GGT CCG CGC TAA GCC GCA TTC AGC CTT CGG
CAA AAT CTC CGC ATG TGG A-- TTT
TTT TTT TCG GCC GGC GTG CGA CCG ATC CGC GAG CCC CTC CAC ATT TTT GCC ACT
CGT GTT CTT CTC TGC GAG CAT AAG
CTA ACT GCG GCT ATT ATA GGA AGC CGC TGA GCT CGG --- ---
>KF951999:_Brassica_rapa_CBS_225.33_Sierra_leone_TEF
--G AGA AGG TAA ACA CAC TTT TCG CAC TCC TGC GCA TGA GGC TGT CGC TGC ACC
AGA ATT TGC CGT ACC TCA TTG ATT
GAG GGG CA- TTT TTT GGT GGG GTT GGT CCG CGC TAA GCC GCA TTC AGC CTT CGG
CAA AAT CTC CGC ATG TGG ATT TTT
TTT TTT TCG GCC GGC GTG CGA CCG ATG CGC GAG CCC CTC CAC ATT TTT GCC ACT
CGT GTT CTT CTC TGC GAG CAT AAG
CTA ACT GCG GCT ATT ATA GGA AGC CGC TGA GCT CGG --- ---
>KF952002:_Cajanus_indicus_CBS_229.33_Sri_Lanka_TEF
--G AGA AGG TAA ACA CAC TTT TCG CAC TCC TGC ACA TGA GGC TGT CGC TGC ACC
AGA ATT TGC CGT ATC TCA TTG ACT
GAG GGG CA- TTT TTT GGT GGG GTT GGT CCG CGC TAA GCC GCA TTC AGC CTT CGG
CAA AAT CTC CGC ATG TGG A-- TTT
TTT TTT TCG ACC GGC GTG CGA CCG ATG CGC GAG CCC CTC CAC ATT TTT GCC ACT
CGT GTT CTT CTC TGC GAG CAT AAG
CTA ACT GCG GCT ATT ATA GGA AGC CGC TGA GCT CGG --- ---
>KF952003:_Gossypium_herbaceum_CBS_230.33_Sudan_TEF
--G AGA AGG TAA ACA CAC TTT TCG CAC TCC TGC ACA TGA GGC TGT CGC TGC ACC
AAA ATT TGC CGT ATC TCA TTG ACT

GAG GGG CA- TTT TTT GGT GGG GTT GGT CCG CGC TAA GCC GCA TTC AGC CTT CGG
CAA AAT CTC CGC ATG TGG A-- TTT
TTT TTT TCG ACC GGC GTG CGA CCG ATG CGC GAG CCC CTC CAC ATT TTT GCC ACT
CGT GTT CTT CTC TGC GAG CAT AAG
CTA ACT GCG GCT ATT ATA GGA AGC CGC TGA GCT CGG --- ---
>KF952004:_Saccharum_officinarum_CBS_231.33_India_TEF
--G AGA AGG TAA ACA CAC TTT TCG CAC TCC TGC ACA TGA GGC TGT CGC TGC ACC
AAA ATT TGC CGT ATC TCA TTG ACT
GAG GGG CA- TTT TTT GGT GGG GTT GGT CCG CGC TAA GCC GCA TTC AGC CTT CGG
CAA AAT CTC CGC ATG TGG A-- TTT
TTT TTT TCG ACC GGC GTG CGA CCG ATG CGC GAG CCC CTC CAC ATT TTT GCC ACT
CGT GTT CTT CTC TGC GAG CAT AAG
CTA ACT GCG GCT ATT ATA GGA AGC CGC TGA GCT CGG --- ---
>KF952005:_Vigna_sinensis_CBS_270.34_Missouri_USA_TEF
--G AGA AGG TAA ACA CAC TTT TCG CAC TCC TGC ACA TGA GGC TGT CGC TGC ACC
AGA ATT TGC CGT ATC TCA TTG ACT
GAG GGG CA- TTT TTT GGT GGG GTT GGT CCG CGC TAA GCC GCA TTC AGC CTT CGG
CAA AAT CTC CGC ATG TGG A-- --T
TTT TTT TCG ACC GGC GTG CGA CCG ATG CGC GAG CCC CTC CAC ATT TTT GCC ACT
CGT GTT CTT CTC TGC GAG CAT AAG
CTA ACT GCG GCT ATT ATA GGA AGC CGC TGA GCT CGG --- ---
>KF952006:_Chrysanthemum_sp._CBS_271.34_Missouri_USA_TEF
--G AGA AGG TAA ACA CAC TTT TCG CAC TCC TGC ACA TGA GGC TGT CGC TGC ACC
AGA ATT TGC CGT ATC TCA TTG ACT
GAG GGG CA- TTT TTT GGT GGG GTT GGT CCG CGC TAA GCC GCA TTC AGC CTT CGG
CAA AAT CTC CGC ATG TGG A-- TTT
TTT TTT --G ACC GGC GTG CGA CCG ATG CGC GAG CCC CTC CAC ATT TTT GCC ACT
CGT GTT CTT CTC TGC GAG CAT AAG
CTA ACT GCG GCT ATT ATA GGA AGC CGC TGA GCT CGG --- ---
>KF952007:_Sorghum_sp._CBS_313.51_Venezuela_TEF
--G AGA AGG TAA ACA CAC TTT TCG CAC TCC TGC ACA TGA GGC TGT CGC TGC ACC
AGA ATT TGC CGT ATC TCA TTT ACT
GAG GGG CA- TTT TTT GGT GGG GTT GGT CCG CGC TAA GCC GCA TTC AGC CTT CGG
CAA AAT CTC CGC ATG TGG A-- -TT
TTT TTT TCG ACC GGC GTG CGA CCG ATG CGC GAG CCC CTC CAC ATT TTT GCC ACT
CGT GTT CTT CTC TGC GAG CAT AAG
CTA ACT GCG GCT ATT ATA GGA AGC CGC TGA GCT CGG --- ---
>KF952008:_Arachis_hypogea_CBS_416.62_Portugal_TEF
--G AGA AGG TAA ACA CAC TTT TCG CAC TCC TGC ACA TGA GGC TGT CGC TGC ACC
AGA ATT TGC CGT ATC TCA TTG ACT
GAG GGG CA- TTT TTT GGT GGG GTT GGT CCG CGC TAA GCC GCA TTC AGC CTT CGG
CAA AAT CTC CGC ATG TGG A-- TTT
TTT TTT TCG ACC GGC GTG CGA CCG ATG CGC GAG CCC CTC CAC ATT TTT GCC ACT
CGT GTT CTT CTC TGC GGG CAT AAG
CTA ACT GCG GCT ATT ATA GGA AGC CGC TGA GCT CGG --- ---
>KF952009:_Phaseolus_aureus_CBS_457.70_Denmark_TEF
--G AGA AGG TAA ACA CAC TTT TCG CAC TCC TGC ACA TGA GGC TGT CGC TGC ACC
AGA ATT TGC CGT A-- TCG TTG ACT
GAG GGG CA- TTT TTT GGT GGG GTT GGT CCG CGC TAA GCC GCA TTC AGC CTT CGG
CAA AAT CTC CGC ATG TGG A-- TTT
TTT TTT TCG ACC GGC GTG CGA CCG ATG CGC GAG CCC CTC CAC ATT TTT GCC ACT
CGT GTT CTT CTC TGC GAG CAT AAG
CTA ACT GCG GCT ATT ATA GGA AGC CGC TGA GCT CGG --- ---
>KF952012:_Glycine_max_CBS_460.70_Denmark_TEF
--G AGA AGG TAA ACA CAC TTT TCG CAC TCC TGC ACA TGA GGC TGT CGC TGC ACC
AGA ATT TGC CGT ATC TCA TTG ACT
GAG GGG CA- TTT TTT GGT GGG GTT GGT CCG CGC TAA GCC GCA TTC AGC CTT CGG
CAA AAT CTC CGC ATG TGG A-- TTT
TTT TTT TCG ACC GGC GTG CGA CCG ATG CGC GAG CCC CTC CAC ATT TTT GCC ACT
CGT GTT CTT CTC TGC GAG CAT AAG

CTA ACT GCG GCT ATT ATA GGA AGC CGC TGA GCT CGG --- ---
>KF952013:_Phaseolus_vulgaris_CBS_461.70_Denmark_TEF
--G AGA AGG TAA ACA CAC TTT TCG CAC TCC TGC ACA TGA GGC TGT CGC TGC ACC
AGA ATT TGC CGT ATC TCA TTG ACT
GAG GGG CA- TTT TTT GGT GGG GTT GGT CCG CGC TAA GCC GCA TTC AGC CTT CGG
CAA AAT CTC CGC ATG TGG A-- TTT
TTT TTT TCG ACC GGC GTG CGA CCG ATG CGC GAG CCC CTC CAC ATT TTT GCC ACT
CGT GTT CTT CTC TGC GAG CAT AAG
CTA ACT GCG GCT ATT ATA GGA AGC CGC TGA GCT CGG --- ---
>KF952018:_Vigna_unguiculata_CPC_11056_Niger_TEF
--G AGA AGG TAA ACA CAC TTT TCG CAC TCC TGC ACA TGA GGC TGT CGC TGC ACC
AGA ATT TGC CGT ATC TCA TTG ACT
GAG GGG CA- TTT TTT GGT GGG GTT GGT CCG CGC TAA GCC GCA TTC AGC CTT CGG
CAA AAT CTC CGC ATG TGG A-- TTT
TTT TTT TCG ACC GGC GTG CGA CCG ATG CGC GAG CCC CTC CAC ATT TTT GCC ACT
CGT GTT CTT CTC TGC GAG CAT AAG
CTA ACT GCG GCT ATT ATA GGA AGC CGC TGA GCT CGG --- ---
>KF952021:_Vigna_unguiculata_CPC_11059_Niger_TEF
--G AGA AGG TAA ACA CAC TTT TCG CAC TCC TGC ACA TGA GGC TGT CGC TGC ACC
AGA ATT TGC CGT ACC TCA TTG ACT
GAG GGG CA- -TT TTT GGT GGG GTT GGT CCG CGC TAA GCC GCA TTC AGC CTT CGG
CAA AAT CTC CGC ATG TGG A-- TTT
TTT TTT TCG GCC GGC GTG CGA CCG ATC CGC GAG CCC CTC CAC ATT TTT GCC ACT
CGT GTT CTT CTC TGC GAG CAT AAG
CTA ACT GCG GCT ATT ATA GGA AGC CGC TGA GCT CGG --- ---
>KF952025:_Vigna_unguiculata_CPC_11065_Niger_TEF
--G AGA AGG TAA ACA CAC TTT TCG CAC TCC TGC ACA TGA GGC TGT CGC TGC ACC
AGA ATT TGC CGT ACC TCA TTG ACT
GAG GGG CA- -TT TTT GGT GGG GTT GGT CCG CGC TAA GCC GCA TTC AGC CTT CGG
CAA AAT CTC CGC ATG TGG A-- TTT
TTT TTT TCG GCC GGC GTG CGA CCG ATC CGC GAG CCC CTC CAC ATT TTT GCC ACT
CGT GTT CTT CTC TGC GAG CAT AAG
CTA ACT GCG GCT ATT ATA GGA AGC CGC TGA GCT CGG --- ---
>KF952032:_Vigna_unguiculata_CPC_11072_Niger_TEF
--G AGA AGG TAA ACA CAC TTT TCG CAC TCC TGC ACA TGA GGC TGT CGC TGC ACC
AGA ATT TGC CGT ATC TCA TTG ACT
GAG GGG CA- TTT TTT GGT GGG GTT GGT CCG CGC TAA GCC GCA TTC AGC CTT CGG
CAA AAT CTC CGC ATG TGG A-- TTT
TTT TTT TCG ACC GGC GTG CGA CCG ATG CGC GAG CCC CTC CAC ATT TTT GCC ACT
CGT GTT CTT CTC TGC GAG CAT AAG
CTA ACT GCG GCT ATT ATA GGA AGC CGC TGA GCT CGG --- ---
>KF952035:_Soil_CPC_11076_Senegal_TEFF
--G AGA AGG TAA ACA CAC TTT TCG CAC TCC TGC ACA TGA GGC TGT CGC TGC ACC
AGA ATT TGC CGT ATC TCA TTG ACT
GAG GGG CA- TTT TTT GGT GGG GTT GGT CCG CGC TAA GCC GCA TTC AGC CTT CGG
CAA AAT CTC CGC ATG TGG A-- TTT
TTT TTT TCG ACC GGC GTG CGA CCG ATG CGC GAG CCC CTC CAC ATT TTT GCC ACT
CGT GTT CTT CTC TGC GAG CAT AAG
CTA ACT GCG GCT ATT ATA GGA AGC CGC TGA GCT CGG --- ---
>KF952048:_Soil_CPC_11095_Senegal_TEF
--G AGA AGG TAA ACA CAC TTT TCG CAC TCC TGC ACA TGA GGC TGT CGC TGC ACC
AGA ATT TGC CGT ATC TCA TTG ACT
GAG GGG CA- TTT TTT GGT GGG GTT GGT CCG CGC TAA GCC GCA TTC AGC CTT CGG
CAA AAT CTC CGC ATG TGG A-- TTT
TTT TTT TCG ACC GGC GTG CGA CCG ATG CGC GAG CCC CTC CAC ATT TTT GCC ACT
CGT GTT CTT CTC TGC GAG CAT AAG
CTA ACT GCG GCT ATT ATA GGA AGC CGC TGA GCT CGG --- ---
>KF952056:_Panicum_miliaceum_CPC_11104_Senegal_TEF
--G AGA AGG TAA ACA CAC TTT TCG CAC TCC TGC ACA TGA GGC TGT CGC TGC ACC
AGA ATT TGC CGT ATC TCA TTG ACT

GAG GGG CA- TTT TTT GGT GGG GTT GGT CCG CGC TAA GCC GCA TTC AGC CTT CGG
CAA AAT CTC CGC ATG TGG A-- TTT
TTT TTT TCG ACC GGC GTG CGA CCG ATG CGC GAG CCC CTC CAC ATT TTT GCC ACT
CGT GTT CTT CTC TGC GAG CAT AAG
CTA ACT GCG GCT ATT ATA GGA AGC CGC TGA GCT CGG --- ---
>KF952065:Panicum_miliaceum_11114_Senegal_TEF
--G AGA AGG TAA ACA CAC TTT TCG CAC TCC TGC ACA TGA GGC TGT CGC TGC ACC
AGA ATT TGC CGT ATC TCA TTG ACT
GAG GGG CA- TTT TTT GGT GGG GTT GGT CCG CGC TAA GCC GCA TTC AGC CTT CGG
CAA AAT CTC CGC ATG TGG A-- TTT
TTT TTT TCG ACC GGC GTG CGA CCG ATG CGC GAG CCC CTC CAC ATT TTT GCC ACT
CGT GTT CTT CTC TGC GAG CAT AAG
CTA ACT GCG GCT ATT ATA GGA AGC CGC TGA GCT CGG --- ---
>KF952067:_Sesamum_indicum_CPC_13080_Mexico_TEF
--G AGA AGG TAA ACA CAC TTT TCG CAC TCC TGC ACA TGA GGC TGT CGC TGC ACC
AGA ATT TGC CGT ATC TCA TTG ACT
GAG GGG CAT TTT TTT GGT GGG GTT GGT CCG CGC TAA GCC GCA TTC AGC CTT CGG
CAA AAT CTC CGC ATG TGG A-- TTT
TTT TTT TCG ACC GGC GTG CGA CCG ATG CGC GAG CCC CTC CAC ATT TTT GCC ACT
CGT GTT CTT CTC TGC GAG CAT AAG
CTA ACT GCG GCT ATT ATA GGA AGC CGC TGA GCT CGG --- ---
>KF952068:_Glycine_max_CPC_13081_Mexico_TEF
--G AGA AGG TAA ACA CAC TTT TCG CAC TCC TGC ACA TGA GGC TGT CGC TGC ACC
AGA ATT TGC CGT ATC TCA TTG ACT
GAG GGG CA- TTT TTT GGT GGG GTT GGT CCG CGC TAA GCC GCA TTC AGC CTT CGG
CAA AAT CTC CGC ATG TGG A-- TTT
TTT TTT TCG ACC GGC GTG CGA CCG ATG CGC GAG CCC CTC CAC ATT TTT GCC ACT
CGT GTT CTT CTC TGC GAG CAT AAG
CTA ACT GCG GCT ATT ATA GGA AGC CGC TGA GCT CGG --- ---
>KF952070:_Sorghum_bicolor_CPC_13082_Mexico_TEF
--G AGA AGG TAA ACA CAC TTT TCG CAC TCC TGC ACA TGA GGC TGT CGC TGC ACC
AGA ATT TGC CGT ATC TCA TTG ACT
GAG GGG CA- TTT TTT GGT GGG GTT GGT CCG CGC TAA GCC GCA TTC AGC CTT CGG
CAA AAT CTC CGC ATG TGG A-- TTT
TTT TTT --G ACC GGC GTG CGA CCG ATG CGC GAG CCC CTC CAC ATT TTT GCC ACT
CGT GTT CTT CTC TGC GAG CAT AAG
CTA ACT GCG GCT ATT ATA GGA AGC CGC TGA GCT CGG --- ---
>KF952073:_Hibiscus_sabdarifa_CPC_21387_Senegal_TEF
--G AGA AGG TAA ACA CAC TTT TCG CAC TCC TGC ACA TGA GGC TGT CGC TGC ACC
AGA ATT TGC CGT ATC TCA TTG ACT
GAG GGG CA- TTT TTT GGT GGG GTT GGT CCG CGC TAA GCC GCA TTC AGC CTT CGG
CAA AAT CTC CGC ATG TGG A-- TTT
TTT TTT TCG ACC GGC GTG CGA CCG ATG CGC GAG CCC CTC CAC ATT TTT GCC ACT
CGT GTT CTT CTC TGC GAG CAT AAG
CTA ACT GCG GCT ATT ATA GGA AGC CGC TGA GCT CGG --- ---
>KF952076:_Vigna_unguiculata_CPC_21392_Senegal_TEF
--G AGA AGG TAA ACA CAC TTT TCG CAC TCC TGC ACA TGA GGC TGT CGC TGC ACC
AGA ATT TGC CGT ATC TCA TTG ACT
GAG GGG CA- TTT TTT GGT GGG GTT GGT CCG CGC TAA GCC GCA TTC AGC CTT CGG
CAA AAT CTC CGC ATG TGG A-- TTT
TTT TTT TCG ACC GGC GTG CGA CCG ATG CGC GAG CCC CTC CAC ATT TTT GCC ACT
CGT GTT CTT CTC TGC GAG CAT AAG
CTA ACT GCG GCT ATT ATA GGA AGC CGC TGA GCT CGG --- ---
>KF952080:_Vigna_unguiculata_CPC_21405_Senegal_TEF
--G AGA AGG TAA ACA CAC TTT TCG CAC TCC TGC ACA TGA GGC TGT CGC TGC ACC
AGA ATT TGC CGT ATC TCA TTG ACT
GAG GGG CA- TTT TTT GGT GGG GTT GGT CCG CGC TAA GCC GCA TTC AGC CTT CGG
CAA AAT CTC CGC ATG TGG A-- TTT
TTT TTT TCG ACC GGC GTG CGA CCG ATG CGC GAG CCC CTC CAC ATT TTT GCC ACT
CGT GTT CTT CTC TGC GAG CAT AAG

CTA ACT GCG GCT ATT ATA GGA AGC CGC TGA GCT CGG --- ---
>KF952120:_Abelmoschus_esculentus_CPC_21470_Senegal_TEF
--G AGA AGG TAA ACA CAC TTT TCG CAC TCC TGC ACA TGA GGC TGT CGC TGC ACC
AGA ATT TGC CGT ATC TCA TTG ACT
GAG GGG CA- TTT TTT GGT GGG GTT GGT CCG CGC TAA GCC GCA TTC AGC CTT CGG
CAA AAT CTC CGC ATG TGG A-- TTT
TTT TTT TCG ACC GGC GTG CGA CCG ATG CGC GAG CCC CTC CAC ATT TTT GCC ACT
CGT GTT CTT CTC TGC GAG CAT AAG
CTA ACT GCG GCT ATT ATA GGA AGC CGC TGA GCT CGG --- ---
>CREA_OF_373.2_Phaseoulus_vulgaris_CREA_OF_373.2_Italy_TEF
--- --- --- --- --- --- --- --- --- --- --- --- --- -GC TGT CGC TGC ACC
AGA ATT TGC CGT ATC TCA TTG ACT
GAG GGG CA- TTT TTT GGT GGG GTT GGT CCG CGC TAA GCC GCA TTC AGC CTT CGG
CAA AAT CTC CGC ATG TGG A-- TTT
TTT TTT --G ACC GGC GTG CGA CCG ATG CGC GAG CCC CTC CAC ATT TTT GCC ACT
CGT GTT CTT CTC TGC GAG CAT AAG
CTA ACT GCG GCT ATT ATA GGA AGC CGC TGA GCT CGG TAA ---
