# Supplementary material for: Occurrence of Macrophomina phaseolina on Chickpea in Italy: Pathogen Identification and Characterization
Source: Pathogens. 2022 Jul 27;11(8):842. doi: 10.3390/pathogens11080842 (PMC9415271; doi:10.3390/pathogens11080842)
Supplement: Supplementary file 1 [file pathogens-11-00842-s001.zip › Supplementary figure 13-06-2022.pptx]

## Slide 1
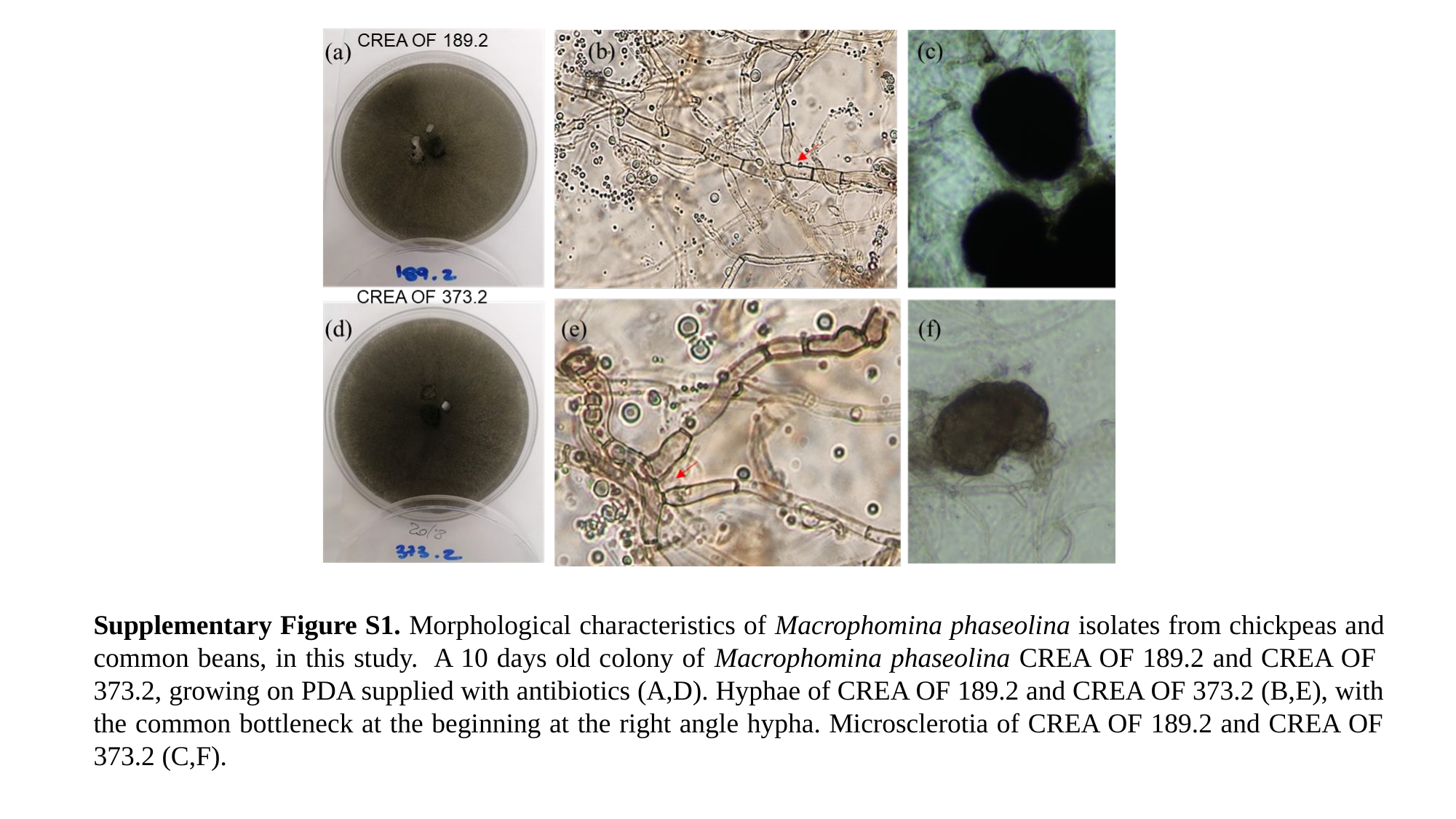

Supplementary Figure S1. Morphological characteristics of Macrophomina phaseolina isolates from chickpeas and common beans, in this study. A 10 days old colony of Macrophomina phaseolina CREA OF 189.2 and CREA OF 373.2, growing on PDA supplied with antibiotics (A,D). Hyphae of CREA OF 189.2 and CREA OF 373.2 (B,E), with the common bottleneck at the beginning at the right angle hypha. Microsclerotia of CREA OF 189.2 and CREA OF 373.2 (C,F).

## Slide 2
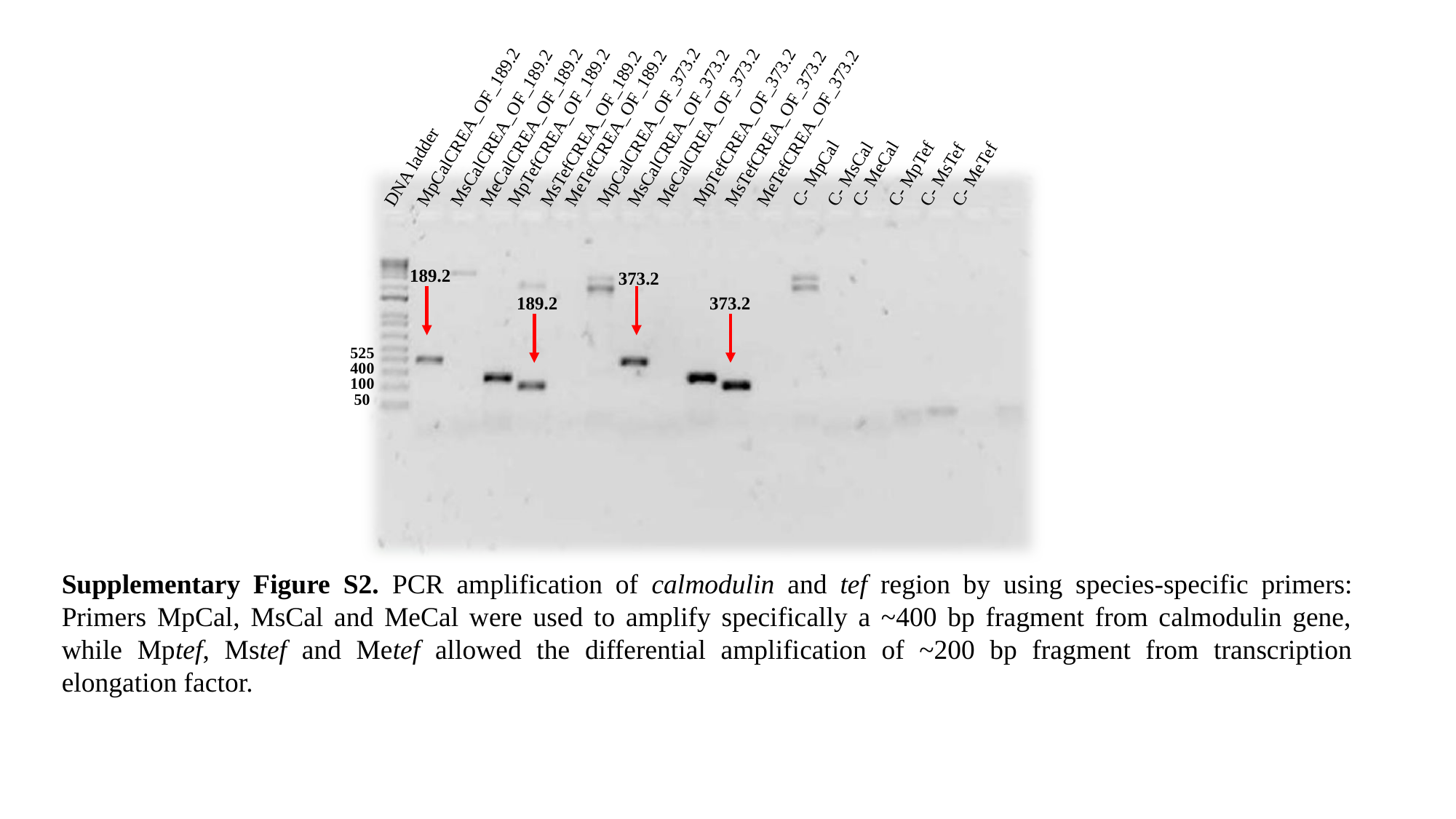

MpTefCREA_OF_373.2
MpCalCREA_OF_189.2
MsCalCREA_OF_373.2
MsTefCREA_OF_189.2
MpCalCREA_OF_373.2
MpTefCREA_OF_189.2
MsTefCREA_OF_373.2
MeTefCREA_OF_189.2
MeCalCREA_OF_373.2
MeCalCREA_OF_189.2
MsCalCREA_OF_189.2
MeTefCREA_OF_373.2
C- MpCal
C- MsCal
C- MeCal
C- MpTef
C- MsTef
C- MeTef
DNA ladder
189.2
373.2
189.2
373.2
525
400
100
50
Supplementary Figure S2. PCR amplification of calmodulin and tef region by using species-specific primers: Primers MpCal, MsCal and MeCal were used to amplify specifically a ~400 bp fragment from calmodulin gene, while Mptef, Mstef and Metef allowed the differential amplification of ~200 bp fragment from transcription elongation factor.

## Slide 3
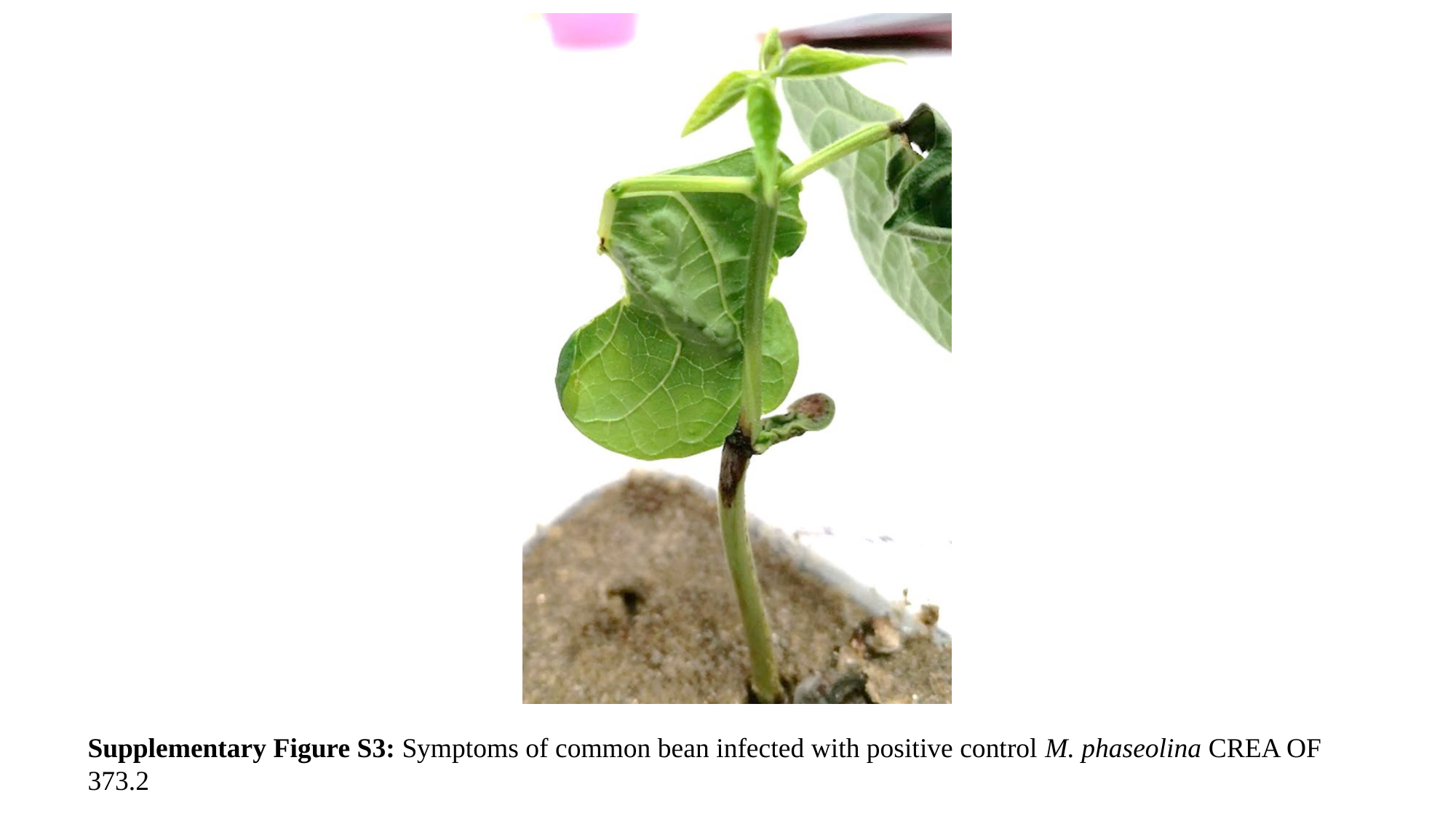

Supplementary Figure S3: Symptoms of common bean infected with positive control M. phaseolina CREA OF 373.2
